# Patient navigators for people with chronic disease: A systematic review

**DOI:** 10.1371/journal.pone.0191980

**Published:** 2018-02-20

**Authors:** Kerry A. McBrien, Noah Ivers, Lianne Barnieh, Jacob J. Bailey, Diane L. Lorenzetti, David Nicholas, Marcello Tonelli, Brenda Hemmelgarn, Richard Lewanczuk, Alun Edwards, Ted Braun, Braden Manns

**Affiliations:** 1 Departments of Family Medicine and Community Health Sciences, University of Calgary, Calgary, Alberta, Canada; 2 Department of Family and Community Medicine, Women’s College Hospital, University of Toronto, Toronto, Ontario, Canada; 3 Department of Medicine, University of Calgary, Calgary, Alberta, Canada; 4 W21C Research and Innovation Centre, Cumming School of Medicine, University of Calgary, Calgary, Alberta, Canada; 5 Department of Community Health Sciences, University of Calgary, Calgary, Alberta, Canada; 6 Faculty of Social Work, University of Calgary, Calgary, Alberta, Canada; 7 Departments of Medicine and Community Health Sciences, University of Calgary, Calgary, Alberta, Canada; 8 Faculty of Medicine and Dentistry, University of Alberta and Alberta Health Services, Edmonton, Alberta, Canada; 9 Department of Family Medicine, Alberta Health Services, Calgary, Alberta, Canada; TNO, NETHERLANDS

## Abstract

**Background:**

People with chronic diseases experience barriers to managing their diseases and accessing available health services. Patient navigator programs are increasingly being used to help people with chronic diseases navigate and access health services.

**Objective:**

The objective of this review was to summarize the evidence for patient navigator programs in people with a broad range of chronic diseases, compared to usual care.

**Methods:**

We searched MEDLINE, EMBASE, CENTRAL, CINAHL, PsycINFO, and Social Work Abstracts from inception to August 23, 2017. We also searched the reference lists of included articles. We included original reports of randomized controlled trials of patient navigator programs compared to usual care for adult and pediatric patients with any one of a defined set of chronic diseases.

**Results:**

From a total of 14,672 abstracts, 67 unique studies fit our inclusion criteria. Of these, 44 were in cancer, 8 in diabetes, 7 in HIV/AIDS, 4 in cardiovascular disease, 2 in chronic kidney disease, 1 in dementia and 1 in patients with more than one condition. Program characteristics varied considerably. Primary outcomes were most commonly process measures, and 45 of 67 studies reported a statistically significant improvement in the primary outcome.

**Conclusion:**

Our findings indicate that patient navigator programs improve processes of care, although few studies assessed patient experience, clinical outcomes or costs. The inability to definitively outline successful components remains a key uncertainty in the use of patient navigator programs across chronic diseases. Given the increasing popularity of patient navigators, future studies should use a consistent definition for patient navigation and determine which elements of this intervention are most likely to lead to improved outcomes.

**Trial registration:**

PROSPERO #CRD42013005857

## Introduction

Chronic diseases, including physical and mental illnesses, are a significant burden to both patients and the health care system. There were an estimated 12.7 million new cases of cancer worldwide in 2008 [1]; and diabetes prevalence was estimated to be 6.4%, affecting 285 million adults worldwide in 2010 [2]. People with chronic diseases have increased morbidity and consume substantially more health care resources than those without [3, 4]. Adherence to evidence-based recommendations for clinical care is associated with better outcomes and lower resource use for patients with chronic diseases [5–7]. For example, clinical trials show that in patients with diabetes, tight control of blood pressure, use of statins and achieving good glycemic control improves outcomes and lowers costs [5]. Despite widespread dissemination of practice guidelines, many people with chronic diseases do not receive or adhere to recommended care [8–11].

This difficulty in implementing evidence-based care may be due to a combination of patient, provider and system level barriers [12]. Patient level barriers may include lack of awareness of publicly funded programs (including community-based resources), financial constraints, competing priorities (e.g., family and work), personal circumstances, language and culture (i.e., race/ethnicity) [13]; such barriers could make it challenging to follow even seemingly simple lifestyle recommendations. At the provider level, barriers may include lack of clinical decision support systems to implement recommended care, lack of time and knowledge. System level barriers include the inherent complexity of the health care system and suboptimal access to primary or specialty care.

Patient navigators are trained personnel who help patients overcome modifiable barriers to care and achieve their care goals by providing a tailored approach to addressing individual needs [14–16]. Navigators may be nurses, social workers or lay health workers, including peers. Patient navigator programs were originally established to reduce gaps in timely cancer care among marginalized populations [17], and are increasingly in use across the United States and Canada within the cancer field [18]. Patient navigation is also currently used for diabetes [19], smoking cessation [20, 21] and cancer screening [22]. Depending on the targeted barriers, specific tasks may include one or more of: disease education [23, 24], health system education [23, 25, 26], removal of medical system barriers [17], assistance with insurance coverage [27], addressing other financial barriers [17], aiding in care coordination [23], referral to community resources [24], and providing emotional support, among others.

Previous reviews of patient navigators have focused on cancer care [16, 28], though patient navigators are increasingly being utilized in other areas [29, 30]. While their popularity is growing, and many are touting their benefits [31], it is not clear whether patient navigator programs are beneficial across a cross-section of chronic diseases. As there is wide variation in the design and implementation of patient navigator programs in various chronic diseases, a systematic review is needed to summarize the characteristics of programs and their effectiveness. In this systematic review, we assess the effectiveness and attributes of patient navigator services, compared with usual care, on patient-oriented outcomes and processes of care in patients with chronic diseases.

## Methods

### Data sources and searches

We searched MEDLINE, EMBASE, The Cochrane Central Register of Controlled Trials (CENTRAL), CINAHL, PsycINFO, and Social Work Abstracts up to August 23, 2017 with no language or date restrictions. In our MEDLINE search strategy, we included potential synonyms for patient navigator (case management, care coordination, health coach), terms for the set of chronic diseases of interest, and restricted the search to randomized controlled trials (S1 File). The MEDLINE search strategy was peer reviewed via PRESS [32]. We also systematically searched reference lists of included studies and relevant reviews.

### Study selection

We included randomized controlled trials (RCTs) evaluating the effectiveness of a patient navigator program compared to usual care. Study population could be adult or pediatric patients, that either had or were being screened for one of the following chronic diseases, as included in the Statistics Canada Canadian Community Health Survey [33]: asthma, arthritis, hypertension, migraine, COPD/emphysema, diabetes, heart disease, cancer, intestinal/stomach ulcers, stroke, urinary incontinence, inflammatory bowel disorder, dementia, mood disorders, anxiety disorders; with the addition of HIV/AIDS, and chronic kidney disease, which includes transplant recipients and patients on dialysis.

There is currently no standard definition of a patient navigator, thus there is variability between patient navigator programs, as well as overlap with programs under different names [34]. We defined a patient navigator as a person with or without a healthcare-related background that engages with patients on an individual basis to determine barriers to accessing care or following recommended guidelines. The patient navigator also provides information relevant to patients’ specific circumstances to facilitate self-management and access to care. We were flexible in terms of the name of the intervention used by study authors (i.e., patient navigator, community health worker, etc.), as long as program descriptions were consistent with our definition. Studies were excluded if they evaluated programs where patient navigators performed clinical care (i.e., prescribed medication, ordered diagnostic tests, performed physical measurements), or where the role was not formalized (i.e., casual or untrained support).

Two reviewers independently screened all titles and abstracts of retrieved references. Two authors then applied the full set of inclusion and exclusion criteria to all articles chosen for full text review. Reviewers resolved any disagreements by discussion.

### Data extraction and quality assessment

Data extraction was done by one reviewer, using standard data extraction forms and verified by a second reviewer. For studies with several trial arms, data were collected across each relevant comparison. Data elements included characteristics of the study, outcomes and results, along with details of the navigator program. Outcome measures of interest fell into one of three broad categories: patient-oriented (mortality, health-related quality of life, and complications of disease, e.g., MI, stroke); surrogate outcomes (e.g., achieving target blood pressure or glycemic control); and process measures, including access to appropriate services, and adherence to recommended clinical actions (e.g., cancer screening). Measures of patient experience and patient satisfaction were also collected. The risk of bias criteria suggested by the Cochrane Effective Practice and Organisation of Care Group (EPOC) were used to assess study quality [33]. Items retained from the tool to assess bias were: random sequence generation, allocation concealment, blinding of outcome assessment, incomplete outcome data, group similarity at baseline and intention-to-treat analysis. Risk of bias in each domain was assessed as high, low or unclear.

### Data synthesis and analysis

Though the goal of the systematic review was to provide a quantitative assessment of the effects of an intervention, we found a heterogeneous group of programs, chronic diseases and outcomes, and we therefore used a narrative approach to data synthesis. To assist in assessing effectiveness across this large number of studies, we tabulated the primary outcome of each of the studies, a summary of the result, and whether the changes were statistically significant. We determined the proportion of studies with positive outcome results (primary or secondary) in each outcome category, stratified by chronic disease. We explored the association between program features and a statistically significant improvement using logistic regression.

Given the heterogeneity in outcomes, it was not possible to assess publication bias using a traditional funnel plot. To provide an estimate of publication bias, we divided the studies into quintiles of sample size and compared the proportion of studies reporting a positive statistically significant effect across quintiles. All work aligned with a protocol that was developed and published ahead of the review [35].

## Results

### Description of studies

**Fig 1:**
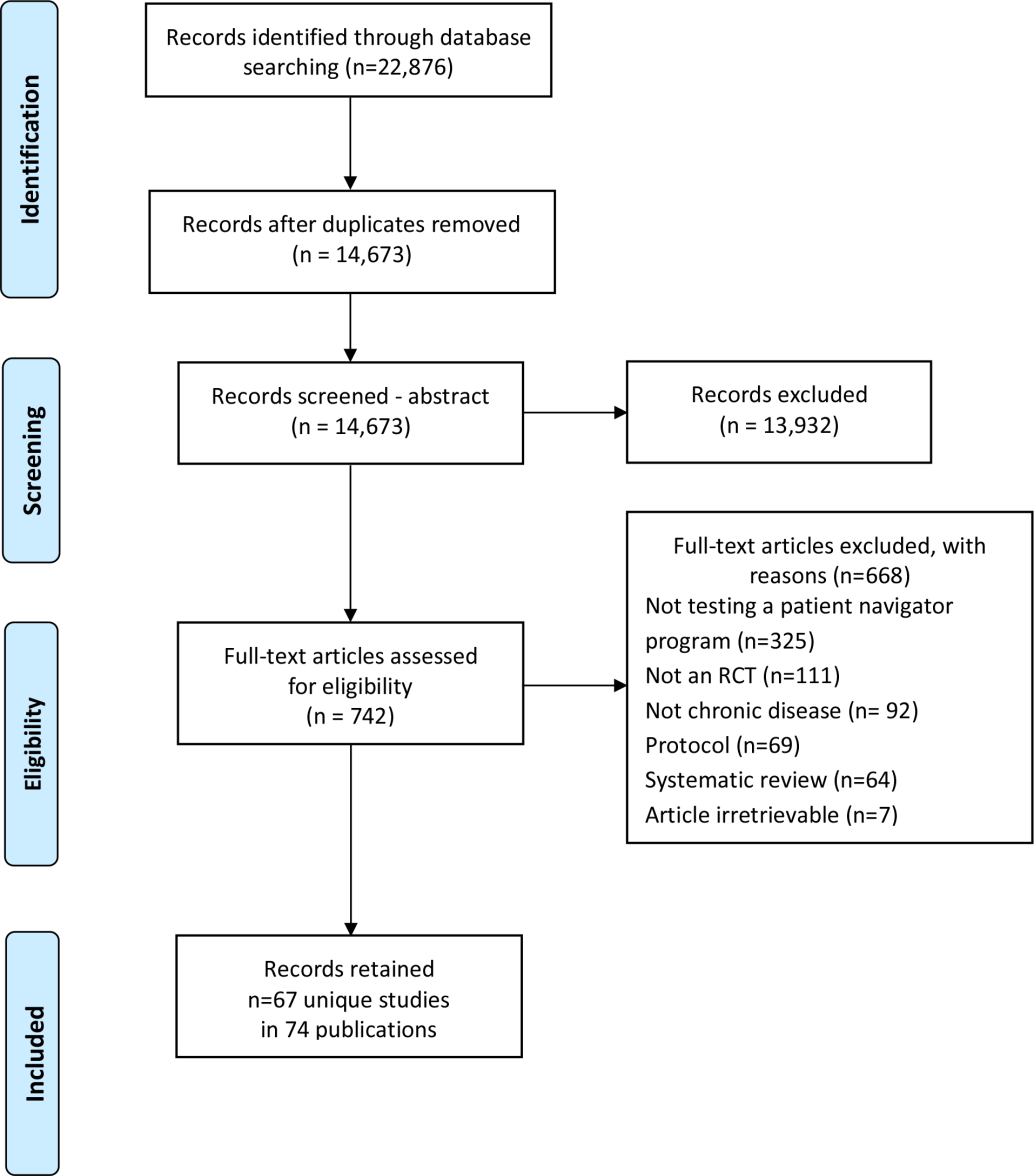
PRISMA flow diagram of study selection.

**Fig 2:**
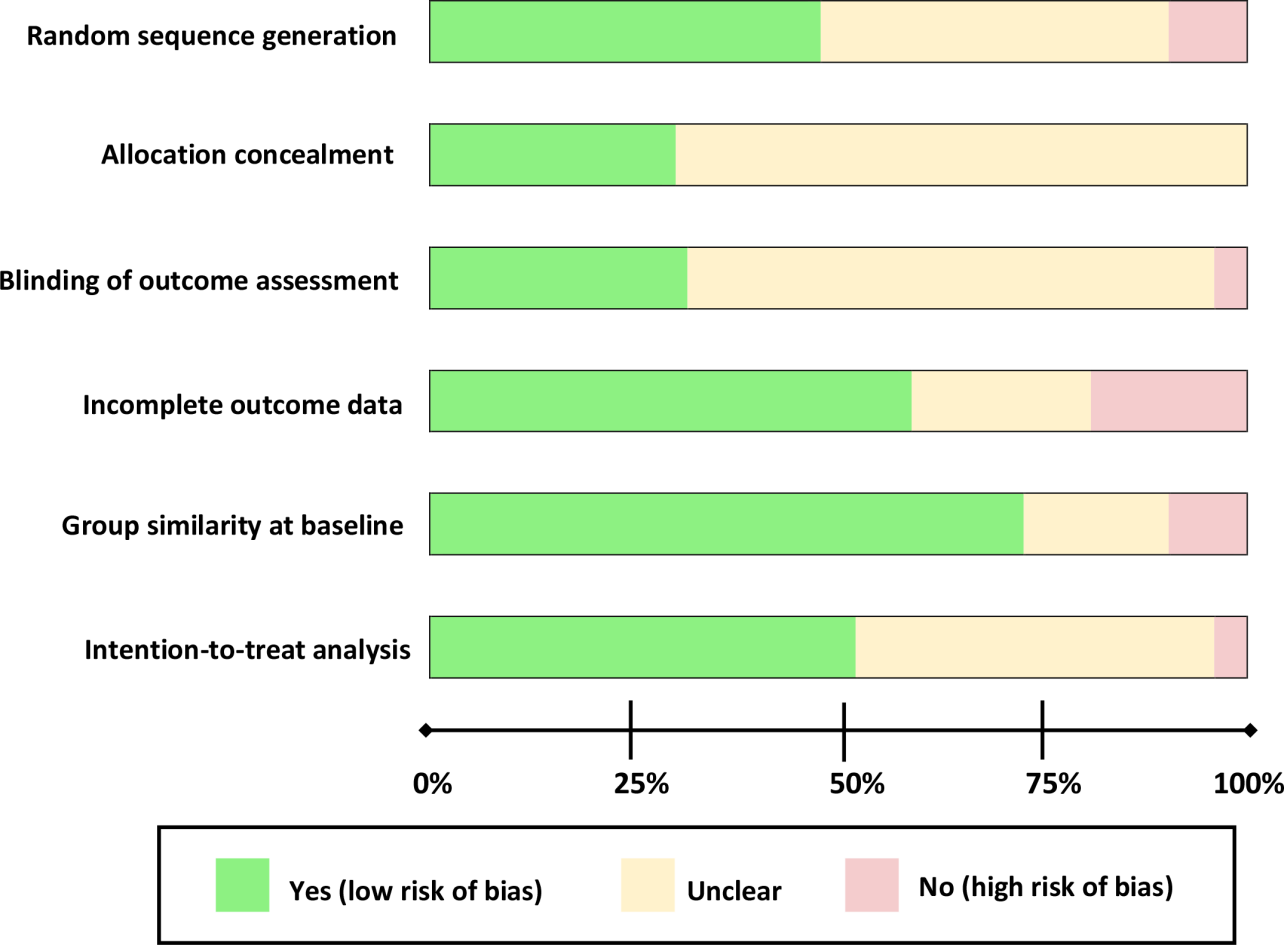
Summary of risk of bias across studies.

We identified and screened 14,672 potentially relevant abstracts. Seventy-four papers describing 67 unique studies met our inclusion criteria, and were included in the review (Fig 1). Table 1 summarizes the characteristics of the included studies, while Table 2 provides an overview of the individual studies, grouped by disease. The vast majority of studies (90%) were conducted in the United States and sample size varied from 21 to 16,267 participants, with the majority of studies (52%) including between 100 and 500 participants. A summary of the quality assessment is presented in Fig 2 and a detailed assessment by study is presented in the Supplementary Table (S1 Table). Though all studies were RCTs, quality varied, and many studies were lacking information on allocation concealment and blinding of outcome assessment.

**Table 1:** Summary of characteristics of included studies.

Characteristic	No. of studies (%) (n = 67)
Country*	
USA	60 (90)
Canada	3 (4)
Australia	2 (3)
UK	1 (1)
France	1 (1)
South Africa	1 (1)
**Disease**	
Cancer	6 (9)
Cancer diagnosis	11 (16)
Cancer screening	27 (40)
Diabetes	8 (12)
CVD	4 (6)
HIV/AIDS	7 (10)
CKD	2 (3)
Dementia	1 (1)
Multiple	1 (1)
**Size**	
<100	7 (10)
100–499	35 (52)
500–999	12 (18)
> = 1000	13 (19)
**Patient Setting**	
Primary care	26 (39)
Specialty clinic	19 (28)
Hospital	7 (10)
Community	12 (18)
Other	3 (4)
**Navigator type**	
Lay	43 (64)
Peer	6 (9)
Professional	8 (12)
Professional + Lay	3 (4)
Not reported	7 (10)
Program features**	
Facilitates care (referrals, communication, coordination)	40 (60)
Appointment scheduling	32 (48)
Accompaniment	17 (25)
Practical support (transportation, dependent care, finance)	39 (58)
Language support	27 (40)
Culturally tailored/ethnicity-matched	28 (42)
Promotes health literacy	29 (43)
Addresses attitudes and beliefs	43 (64)
Encourages treatment adherence	13 (19)
Appointment reminders	34 (51)
Delivers education	58 (87)
Provides psychosocial support	42 (63)
Communication medium**	
Telephone	60 (90)
In-person—patient home	18 (27)
In-person—other	38 (57)
Mail	32 (48)
E-mail	6 (9)
**Intensity—frequency**	
Brief (1–2 contacts, ≤1 in-person)	19 (28)
Medium (2–5 contacts)	15 (22)
High (contact as needed)	33 (49)
**Intensity—duration**	
Up to 3 months	21 (31)
3–12 months	34 (51)
>12 months	12 (18)

*one study conducted in USA and Canada

**not mutually exclusive

**Table 2:** Summary of included studies.

Author(s) Year Country	Size	Population and setting	Intervention	Control	Max duration of navigation (follow-up if differs)	Outcomes (primary bolded)	Summary of primary outcome effect—bolded if significant (intervention vs control)
**Cancer treatment**							
Fiscella/ Hendren 2012 USA [36, 37]	438	Underserved men or women with newly diagnosed breast or colorectal cancer at two outpatient study sites (Rochester and Denver)	Face-to-face assessment with PN to identify and address barriers, provide education and emotional support	Usual care	12 months	**Time to completion of adjuvant treatment** Psychological distress; satisfaction with cancer care; QOL (in subset of 319 Rochester patients)	57 vs 63 days (p>0.05)
Ell 2009 USA [38]	487	Low-income women with newly diagnosed breast or gynecological cancer at oncology practices	Telephone assessment with PN to assess barriers, and tiered navigation services across three levels of intensity based on need	Usual care + written info on available resources and education pamphlet	12 months	**Adherence to adjuvant treatment**	No SS difference
White 2012 Australia [39]	653	Patients with newly diagnosed colorectal cancer in the community	Letter with feedback on unmet needs and levels of anxiety and depression; Pathfinder provided emotional, informational, and instrumental support with written action plan and follow-up calls; relay to primary care physician	Usual care	12 months	**Change in supportive care needs; Change in anxiety & depression** Colorectal symptoms; Use of support services; Social support	No SS difference in supportive care needs; **decrease in anxiety (p<0.001**)
Percac-Lima 2015 USA [40]	3234	Outpatients with cancer at high predicted risk of missing a scheduled appointment at oncology practices	PN provided telephone appointment reminders, reviewed upcoming visit procedures, identified and addressed barriers, facilitated communication with members of practice	Usual care	1 week (5 months)	**Failure to attend appointment** Number of vacated timeslots filled	**10.2% vs 17.5% (p<0.001)**
Giese-Davis 2016 USA [41]	104	English-speaking patients aged 20–85 diagnosed with breast cancer in the last 3 months in the community	Related stories, provided support, connected to community resources, recognize trauma symptoms, and assess need for professional help	Usual care	6 months	**Quality of life; Cancer-related trauma symptoms** Cancer self-efficacy; Marital interaction; Depression symptoms; Breast cancer resources	**QOL: SS higher (p = 0.01)**; no SS difference in trauma symptoms
Shaw 2016 Australia [42]	128	Caregivers and adult patients discharged home from surgical treatment for advanced gastrointestinal cancers	Telephone based assessment of caregiver well-being, barriers to care, and needs, connection with local resources	Usual care	10 weeks (6 months)	**Caregiver quality of life** Caregiver: Burden; Psychological distress; Unmet needs; Patient: Acute care utilization; QoL; Unmet needs; Psychological distress	No SS difference
**Cancer diagnosis**							
**Breast cancer**							
Ferrante 2008 USA [43]	105	Low-income and minority women with abnormal mammogram findings at outpatient hospital clinic	Needs assessment and tailored navigation by PN, including emotional support, appointment scheduling and preparation, reminders, financial applications, access to care/resources, facilitated communication with health care team	Usual care	2 months	**Time to diagnostic resolution** Change in anxiety; Patient satisfaction	**25.0 vs 42.7 mean days (p = 0.001)**
Ell 2007 USA [44]	204	Low-income women with abnormal mammogram findings at public medical clinic	PN provided adherence risk assessment, health education and psychosocial counseling, systems navigation, tracking and reminders, referral to community resources, according to 3 tiered service levels based on assessed risk	Usual care	8 months	**Diagnostic resolution within 8 months** Timely follow-up	**90% vs 66% (p<0.001)**
Crump 2008 USA [45]	83	African American women with abnormal mammogram findings at hospital breast clinic	Lay health advocate provided reminders via telephone or mail, education, identified and addressed barriers, referral to resources, accompaniment	Usual care	6 months	**Attendance at follow-up appointment** Attendance at FNA/biopsy if indicated; Attendance at all follow-up appointments	**95.8% vs 77.1% (p = 0.02)**
Maxwell 2010 USA [46]	176	Korean American women with abnormal breast findings who had missed follow-up appointment at community health clinic	PN provided reminders, informational support, education, assistance with completing forms, emotional support, accompaniment	Usual care	6 months	**Completion of follow-up diagnostic exam**	**97% vs 67% (p<0.001)**
Bastani 2010 USA [47]	1671	Low-income and minority women with abnormal breast findings at county hospital outpatient clinics	Professional provided information and education, support and encouragement, and addressed barriers via telephone, followed by regular contact with lay health worker (LHW) to provide informational, emotional and social support, encouragement and health system navigation	Usual care	6 months	**Diagnostic resolution within 6 months**	55% vs 56% (p = 0.56)
**Cancer diagnosis**							
**Cervical cancer**							
Lerman 1992 USA [48]	90	Low-income minority adolescents and women who missed scheduled colposcopy	Telephone counseling to address educational barriers, psychological barriers and practical barriers, using scripted messages	Telephone call to reschedule appointment and assess barriers	Once (NR)	**Attendance at colposcopy appointment**	**67% vs 43% (p<0.05)**
Miller 1997 USA [49]	828	Low-income minority women with an abnormal pap test referred to colposcopy	(1) Pre-colposcopy appointment structured phone call to elicit and address expectancy, emotional, and practical barriers, using scripted messages; (2) Same as 1 and with booster call prior to 6-month follow-up visit	(1) Telephone reminder 1 week ahead of appointment (C+R); (2) No telephone contact (C)	Once (6 months)	**Attendance at first colposcopy appointment within 6 months** Attendance at follow-up colposcopy appointment	**76% vs 68% (p<0.05); OR 1.50 (1.04, 2.17)** (I 1&2 vs C+R)
Engelstad 2005 USA [50]	348	Low-income minority women with an abnormal pap test at county hospital outpatient clinics	Computerized tracking system; In-person assessment with Community Health Advisor and structured counseling, appointment scheduling, reminders, and follow-up for missed appointments	Usual care	6 months	**Attendance at follow-up appointment within 6 months**	**61% vs 32% (p = 0.001)**
**Cancer diagnosis**							
**Multiple types**							
Wells/Lee/Lee 2012 USA [51–53]	1576	Underserved men or women with breast (1294) or colorectal (282) cancer screening abnormality at primary care clinics	PN identified and addressed patient-level barriers, provided education, appointment scheduling	Usual care	6 months	**Change in median time to diagnostic resolution; Change in proportion with diagnostic resolution within 6 months** Median time to diagnostic resolution in breast ca. only; Median time to diagnostic resolution in colon ca. only	42 to 61 days vs 38 to 42 days (p = 0.16); 61.9 to 74.5 vs 68.2 to 68.5 (p = 0.07)
Raich 2012 USA [54]	993	Underserved men or women with breast (628), colorectal (235) or prostate cancer (130) screening abnormality at public safety net clinic	PN identified and addressed patient barriers, encouragement, social support; scheduled exams, communicated with clinical staff; accompaniment	Usual care	12 months	**Time to diagnostic resolution within 12 months** Diagnostic resolution within 12 months; Number of cancers diagnosed; Stage at diagnosis	**Shorter for intervention (p<0.001)** (details not provided)
Paskett 2012 USA [55]	862	Men or women with breast, cervical or colorectal cancer screening abnormality at primary care clinics	PN identified needs, connected with services, facilitated communication with clinical staff, provided education, social support	Mailed educational materials	15 months (NR)	**Time to diagnostic resolution; Diagnostic resolution within timeframe**	**PN more effective over time (p = 0.012); HR increased from 1.17 (0.9, 1.52) to 1.65 (1.16, 2.33) from 3 to 15 months (p = 0.009)**
**Cancer screening**							
**Breast cancer**							
Weber 1997 USA [56]	376	Low-income urban women due for mammogram in primary care	Primary care physician letter, Community Health Educator sent letter, provided outreach with education, reminders, identified and addressed barriers to care using telephone, home/office visits, or mail	Letter from primary care physician informing of need for screening	4 months	**Completion of mammogram** Cost-effectiveness	**25% vs 9.8% (p<0.001); RR 2.57 (1.53, 4.35)**
West 2004 USA [57]	237	Low-income rural African American women due for mammography and who remained non-adherent after 6 months from the community	Community Health Care Workers provided tailored counseling by phone to identify and address barriers, provide personalized education, provided information re. scheduling and financial assistance	Tailored letter to promote adherence to screening	Once (6 months)	**Completion of mammogram within 12 months**	15% vs 13% (p = NS)
Paskett 2006 USA [58]	851	Low-income rural women due for mammogram at rural community health center	Intensive education by Lay Health Advisor via two home visits, identified and addressed barriers, appointment scheduling, follow-up phone calls and mailings	Usual care	12 months	**Completion of mammogram** Cost of intervention; Changes in modifiable factors (barriers, beliefs, knowledge)	**42.5% vs 27.3% (p<0.001); RR 1.56 (1.29, 1.87)**
Rahm 2007 USA [59]	125	Women referred for hereditary breast and ovarian cancer genetic counseling	Telephone contact with PN to provide education about process, appointment scheduling, reminders	Usual care	3 months (9 months)	**Attendance at appointment within 9 months** Time to appointment; Cost	44% vs 31% (p = 0.16)
Ahmed 2010 USA [60]	2357	Low-income women due for mammogram in managed care organization	Stepped-care intervention: 1) reminder letter; 2) letter from primary care physician, and 3) face-to-face counseling with Community Health Outreach Worker that was tailored to need with a focus on education, fears, and resources	(1) Usual care (C); (2) Usual care + reminder letter (C+L)—both had self-referral access to outreach workers	Once (12 months)	**Completion of mammogram**	**27% (I) vs 16% (C+L) vs 13% (C) (p<0.01); RR 2.03 (1.64, 2.51) c/w C, 1.69 (1.39, 2.06) c/w C+L**
Phillips 2011 USA [61]	3895	Low-income women eligible for mammogram at academic primary care clinic	Telephone contact with PN for women with no mammogram in previous 18 months (661) to identify and address barriers, schedule appointments; integrated with primary care team and interacted with providers	Usual care	9 months	**Completion of mammogram within 2 years** Adherence rates by interval from last mammogram	**87% vs 76% (p<0.001); OR 2.5 (1.9, 3.2)**
Marshall 2016 USA [62]	1705	African American women > = 65 eligible for mammogram in primary care	Educational materials + in-person/telephone support with PN who identified and addressed barriers, appointment scheduling, education, accompaniment, coaching on communication with providers	Printed educational materials	24 months (up to 46 months)	**Completion of mammogram within 2 years**	**93.3% vs 87.5% (p<0.001); OR 2.26 (1.59, 3.22)**
**Cancer screening**							
**Cervical cancer**							
Taylor 2002 USA/ Canada [63]	482	Chinese American women due for pap test from the community	Home visit by Outreach Worker with educational materials, watched video together and provided tailored counseling and social and logistical support, with follow telephone contact	(1) Usual care (C); (2) Mail intervention with video and printed educational materials (C+E)	1 month (6 months)	**Completion of Pap test within 6 months and within 2 years**	**6 months: 39% vs 15% (p<0.001 for I vs C); OR 3.5 (1.9, 6.6); 2 years: 61% vs 34% (p<0.001 for I vs C); OR 3.3 (1.9, 5.7)**
Taylor 2010 USA [64]	234	Vietnamese American women due for pap test from the community	Culturally appropriate educational materials including video, home visits by Lay Health Worker, social support, education, follow-up via telephone	Educational materials on physical activity	1 month (6 months)	**Completion of Pap test within 6 months**	24% vs 14% (p = 0.07)
**Cancer screening**							
**Colorectal cancer**							
Jandorf 2005 USA [65]	78	Low-income minority men or women due for colorectal cancer screening in primary care	Same as control plus PN who provided reminders, education, appointment scheduling, support and encouragement via telephone	FOBT cards placed in charts and physicians recommended screening	6 months	**Completion of FOBT within 3 months; Completion of endoscopy within 6 months**	42.1% vs 25% (p = 0.086); **23.7% vs 5% (p = 0.019)**
Basch 2006 USA [66]	456	Men or women due for colorectal screening; majority black race from the community	Education via telephone outreach, motivational counseling, addressed barriers, social and emotional support	Printed educational materials	6 months	**Completion of CRC screening within 6 months**	**27.0% vs 6.1% (p = NR); RR 4.4 (2.6, 7.7)**
Percac-Lima 2008 USA [67]	1223	Low-income and minority men or women due for colorectal cancer screening in primary care	Mailed educational materials, telephone or in-person contact with PN to provide education and identify and address barriers, tailored follow-up contact with appointment scheduling, reminders, and accompaniment	Usual care—no PN	9 months	**Completion of CRC screening within 9 months** Completion of colonoscopy specifically; Number of polyps and cancers per 100 patients	**27.4% vs 11.9% (p<0.001)**
Christie 2008 USA [68]	21	Low-income minority men or women due for colorectal cancer screening at community health center	PN facilitated referral for colonoscopy, telephone contact to provide education, appointment scheduling, identified and addressed barriers, follow-up	Referral form placed in patient chart for physician to complete	6 months	**Completion of CRC screening at 3 and 6 months** Colonoscopy refusal; quality of colon prep	53.8% vs 13% (p = 0.085)
Lasser 2011 USA [69]	465	Low-income men or women due for colorectal cancer screening in primary care	Letter from primary care provider and printed materials + telephone contact from PN to provide education, identify and address barriers, counselling tailored to stage of change, facilitate referral for colonoscopy and appointment scheduling, emotional support	Usual care	6 months (12 months)	**Completion of CRC screening within 1 year** Proportion screened by colonoscopy; Proportion with detected adenomas or cancer; Proportion with high-risk lesions	**33.6% vs 20.0% (p<0.001)**
Coronado 2011 USA [70]	501	Hispanic men or women due for colorectal cancer screening at community health clinic	Mailed FOBT card with instructions +/- telephone call with Promotora to provide education and reminders +/- home visit to provide more details and address misconceptions	(1) Usual care (C); (2) Mailed FOBT cards with instructions (C+M)	9 months	**Completion of FOBT screening** Survey reported awareness, participation, knowledge and attitudes about CRC screening	31% vs 26% (C+M) vs 2% (C) (p<0.001 for I vs C and C+M vs C; p = 0.28 for I vs C+M)
Green 2013 USA [71]	4,675	Men or women due for colorectal cancer screening in primary care	(1) Automated reminders + telephone assistance from medical office assistant to review screening intent and facilitated communication with primary care physician (I-1); (2) Automated reminders + telephone assistance + nurse navigation for patients with questions or seeking FOBT alternative—risk assessment, motivational counseling, assisting with referrals and appointment scheduling (I-2)	(1) Usual care with opportunistic reminders (C); (2) Usual care + automated reminders (C+R)	24 months	**Completion of any CRC screening within 2 years; Being current with CRC in years 1 and 2** Completion of specific types of CRC in year 1 and year 2; Intervention costs	**Any: 82.6% vs 77.9% vs 72.5% vs 57.4%; Current in Y1/Y2: 64.7% vs 57.5% vs 50.8% vs 26.3% (p<0.001 for all)** **(I-2) vs (I-1) vs (C+R) vs (C)**
Myers/ Lairson 2013 USA [72, 73]	945	Men or women due for colorectal cancer screening in primary care	Baseline survey then (1) Mailed booklet and FOBT kit, nurse available to answer questions and provide reminder (SI); (2) Mailed booklet + materials specific to preferred screening method, phone call from Navigator to verify preference, identify and address barriers, provide encouragement, reminders (TNI)	Baseline survey to assess screening attitudes and preferences, then usual care (C)	2 months (12 months)	**Completion of CRC screening within 6 months** Completion of CRC screening within 12 months; Change in overall screening preference at 6 months; Cost-effectiveness	**38% (TNI) vs 33% (SI) vs 12% (p<0.001); OR 4.60 (3.02, 7.02) (TNI vs C), 3.69 (2.42, 5.64) (SI vs C), 1.25 (0.89, 1.75) (TNI vs SI)**
Myers 2014 USA [74]	764	African American men or women due for colorectal cancer screening in primary care	Baseline survey, mailed materials based on preference, personalized message identifying and addressing barriers, telephone contact from Navigator to review materials, address barriers, and follow up, and a reminder letter (TNI)	Baseline survey to assess screening attitudes and preferences, mailed booklet and FOBT kit, phone number to schedule colonoscopy, reminder letter (SI)	2 months (12 months)	**Completion of CRC screening within 6 months** Completion of CRC screening within 12 months; Change in overall screening preference at 6 months	**38.0% vs 23.7% (p = 0.001); OR 2.1 (1.5, 2.9)**
Enard 2015 USA [22]	303	Latino American men or women due for colorectal cancer screening from the community	Mailed materials + telephone contact from PN with structured automated prompts to identify and address barriers, assess screening history and provide education; follow up	Mailed educational materials	6–48 months (up to 48 months)	**Completion of CRC screening—variable follow-up** Completion of specific screening tests	**43.7% vs 32.1% (p = 0.04); OR 1.64 (1.02, 2.62)**
Ritvo 2015 Canada [75]	5240	Men or women due for colorectal cancer screening in primary care	Introductory letter and invitation to in-person or telephone appointment for tailored nurse navigation based on screening preference; included education, facilitation of referrals, appointment scheduling	Usual care	Once (12 months)	**Completion of CRC screening within 12 months**	**35% vs 20% (p<0.001); OR 2.11 (1.87, 2.39)**
Greenspan 2016 USA Abstract **[76]**	155	Men and women referred for colonoscopy screening at a hospital clinic	Telephone contact to address topics related to colonoscopy in a patient-directed manner	Usual care	Twice (NR)	**Attendance at colonoscopy screening;** Adequacy of bowel preparation	Attendance: 76.3% vs 77.3% (p = 0.99; Bowel prep: no SS difference
Cole 2017 USA [77]	731	African American men aged 50 years of older with uncontrolled hypertension and due for colorectal cancer screening	Telephone contact to assess logistic and psychosocial barriers to screening, educate, facilitated colonoscopy appointments, sent FIT screening tests to participants.	Motivational interviewing for blood pressure control	Twice (6 months)	**Completion of CRC screening within 6 months**	**17.5% vs 8.4% (p<0.01)**
Guillame 2017 France [78]	16267	Men and women due for colorectal cancer screening in the community	Invitation letter followed by phone calls, personalized information and assistance to address and overcome barriers (screening population was randomized but intervention was delivered to navigable population- overdue for screening and with valid phone number)	Usual care	Once (9 months)	**Completion of FOBT screening (screening and navigable populations)** Reduction in social inequalities	Screening: 29% vs 27.5, OR 1.08 (0.99, 1.18); **Navigable: 24.3% vs 21.1%, OR 1.19 (1.10, 1.29)**
DeGroff 2017 USA [79]	843	English or Spanish speaking adults from low-income neighbourhoods referred for colonoscopy screening at a hospital clinic	Telephone contact to assess barriers, educate, address emotional concerns, make appointments, arrange transportation services, and facilitated communication with healthcare providers	Usual care	6 months	**Completion of colonoscopy screening within 6 months** Adequacy of bowel preparation	**61.1% vs 53.2 (p = 0.021); OR 1.51 (1.12–2.03)**
**Cancer screening**							
**Multiple types**							
Dietrich 2006 USA [80]	1413	Low-income and minority women 50–69 years of age due for breast, cervical or colorectal cancer screening from community and migrant health centers	Printed materials + telephone support from Prevention Care Manager to identify and address barriers, motivational support using a structured script, appointment scheduling, education, reminders, provider communication tools	Printed educational materials and a single telephone call from trial staff to promote preventive care	18 months	**Completion of cancer screening within defined interval**	43% vs 30% (p = NR)
Braun 2015 USA [81]	488	Asian and Pacific Islanders eligible for breast, cervical, colorectal or prostate cancer screening from the community	Telephone or home visits from PN, identified and addressed barriers, provided education, appointments and reminders, communication with providers	Educational materials on nutrition and cancer	24 months (NR)	**Completion of cancer screening within defined interval**	**Pap test: 57.0% vs 36.4% (p = 0.001); Mammogram: 61.7% vs 42.4% (p = 0.003); PSA test: 54.4% vs 36.0% (p = 0.008); Endoscopy: 43.0% vs 27.3% (p<0.001); FOBT: 20.7% vs 12.6% (p = 0.02)**
Percac-Lima 2016 USA [82]	1612	Patients overdue for at least one screening test and at high risk for not completing screening in primary care	IT enabled patient tracking; telephone contact to explore individual barriers, provide motivational interviewing, reminders, arrange transportation, visit preparation, and accompaniment if needed	Usual care—included electronic/phone reminders	8 months	**Percentage of time screening was up to date during 8 months for all cancers and by individual cancer** Completion of any and each type of cancer screening within 8 months; as treated analysis	**All cancers: 10.2% vs 6.8%; MD 3.4% (1.5, 5.2); Breast: 14.7% vs 11.0%; MD 3.7% (0.2, 7.3); Cervical: 11.1% vs 5.7%; MD 5.4% (2.1, 9.2); Colorectal: 7.6% vs 4.6%; MD 3.0% (0.7, 5.2)**
**Diabetes**							
Corkery 1997 USA [83]	64	Hispanic and African American men or women with type 2 diabetes enrolled in diabetes education program at a diabetes clinic	Enrolled in diabetes education and Community Health Worker acted as liaison, provided accompaniment, served as an interpreter, reinforced care instruction, reminders, appointment scheduling	Enrolled in diabetes education program	6 months (mean 7.7 months)	**Program completion** Change in knowledge-gain scores; Change in A1C	**80% vs 47% (p = 0.01)**
Laffel 1998 USA [84]	171	Adolescents with type 1 diabetes at a pediatric and adolescent diabetes clinic	Care Ambassador provided appointment scheduling, assistance with health insurance/finances, monitor clinical attendance, via telephone	Usual care	24 months	**Frequency of visits; A1C at 24-months; Severe hypoglycemia; ER visits or hospitalizations** Patients with <3 visits per year	**Mean visits: 7.1 vs 5.2 (p = 0.0001);** Mean A1C: 8.6% vs 8.7%; **Hypoglycemia: 10.1 vs 22.5/100 pt-years (p = 0.009); Hospitalizations: 10.6 vs 20.5/100 pt-years (p = 0.034)**
Svoren 2003 USA [85]	299	Children and adolescents with type 1 diabetes at a pediatric and adolescent diabetes clinic	(1) Care Ambassador: Appointment scheduling, assistance with health insurance/finances, monitor clinical attendance, via telephone (CA); CA+: CA + educational modules implemented by CA's (CA+)	Usual care	24 months	**Frequency of visits; A1C at 24-months; Severe hypoglycemia; ER visits or hospitalizations** Patients with <3 visits per year	**Mean visits: 7.3 CA vs 7.5 CA+ vs 5.4 C (p = 0.0001, CA/CA+ vs C)**; A1C: no difference; Hypoglycemia: 56.1 vs 45.4 vs 64.8/100 pt-years; ED visits: 37.5 vs 21.0 vs 32.6/100 pt-yrs; Hospitalizations: 18.2 vs 8.9 vs 12.7/100 pt-yrs
Gary 2004 USA [86]	186	African American men or women with type 2 diabetes in primary care	Home visits or telephone calls with Community Health Worker to provide education, assist with appointment scheduling, monitor adherence, mobilize social support, provide feedback to primary care provider (CHW) (2 additional arms including nurse case management were also part of this study)	Usual care + quarterly newsletter	24 months	**Change in A1C over 2 years** Change in lipid profile and blood pressure; Dietary practices; Physical activity	-0.30 ± 0.48%, CHW c/w C (p = NS)
Spencer 2011 USA [87]	164	African American and Latino men or women with type 2 diabetes in primary care	Group education classes, 2 home visits & 1 clinic visit with Family Health Advocate providing comprehensive education, addressing patient goals, improving communication skills, facilitating referrals, telephone follow-up (FHA)	Usual care—access to community diabetes programming and monthly contact to update information	6 months	**Change in A1C over 6 months** Change in LDL and BP; Self-management knowledge; Self-management; Psychological distress; Self-efficacy; Physical activity and dietary practices	**-0.99% FHA c/w C (p<0.01)**
Thom 2013 USA [19]	299	Low income men or women with type 2 diabetes and A1C > = 8.0% in primary care	In person and telephone contact with Peer Health Coach to assist with care planning, social and emotional support, assist with accessing care/resources, lifestyle and medication adherence	Usual care	6 months	**Change in A1C over 6 months** Proportion with A1C decrease of > = 1.0%; Proportion with A1C <7.5% at 6 months; Change in LDL, SBP, BMI over 6 months	**-1.07% vs -0.3% (p = 0.01)**
Prezio 2013 USA [88]	180	Uninsured Mexican American men or women with type 2 diabetes from a community health center	In-person meetings with Community Health Worker to provide education, facilitated physician contact and pharmacy refills, arranged referrals (CHW)	Printed education materials, free blood glucose monitor and testing strips	12 months	**Change in A1C over 12 months** Number of medication changes; change in lipids, BP, BMI over 12 months	**-0.7% CHW c/w C (p = 0.02)**
Carrasquillo 2014 USA [89]	300	Latino American men or women with type 2 diabetes and A1C > = 8% in primary care	Structured intervention by Community Health Worker with home visits, telephone calls and group education sessions, navigation and assistance with social and non-medical needs	Usual care	12 months	**Change in SBP, LDL, and A1C over 12 months**	**A1C: -0.38 vs +0.21% (p = 0.01)**; SBP -3 vs -1 mm Hg (p = NS); LDL: -1 vs +3 mg/dL (p = NS)
**Cardiovascular risk**							
Willard-Grace/Thom/Thom 2015 USA [90–92]	441	Low income men or women with one of: type 2 diabetes and uncontrolled A1C, uncontrolled BP, or uncontrolled LDL in primary care	In person and telephone contact from Health Coach to provide assistance with self-management skills, emotional and social support, medication and lifestyle adherence, clinic navigation, referrals, appointment scheduling, accompaniment; Contact via in-person medical visits and telephone	Usual care	12 months	**Proportion reaching at least one of A1C, SBP or LDL target among those not at target** Proportion reaching all targets and each of A1C, SBP or LDL; Proportion meeting goals regardless of baseline values; Change in patient trust in PCP and patient recommendation of PCP; Number of visits to PCP over 12 months; Change in medication concordance and medication adherence	**46.4% vs 34.3% (p = 0.02)**
**Cardiovascular disease**							
Scott/Scott 2013 USA [26, 93]	181	Men or women in hospital with an indication for outpatient cardiac rehabilitation	In-person meeting with PN prior to discharge to provide education, facilitate enrolment in outpatient cardiac rehabilitation program, follow up by telephone	Usual care—discharge instructions	2 weeks (3 months)	**Attendance at outpatient cardiac rehabilitation program** OCR awareness at 12 weeks	**23.6% vs 6.7% (p = 0.003)**
Dennis 1997 USA [94]	417	Men or women with recent stroke in hospital and community post-discharge	Family Care Worker identified and addressed patient needs, assist with accessing care/resources, counselling	Usual care	6 months	**Patient and carer reported outcomes across multiple domains, including health status, social adjustment; Patient and carer satisfaction**	No important differences in patient or carer reported outcomes; Satisfaction higher in both patients and carers in intervention group
Ali-Faisal 2016 Canada [95]	94	Adult cardiac inpatients eligible for cardiac rehabilitation after discharge home	In-person visit to encourage enrolment in cardiac rehab and provide written materials, mailed card, phone call to discuss barriers to enrolment	Usual care—included eReferral	2 weeks (3 months)	**Attendance at cardiac rehabilitation** Referral to cardiac rehabilitation	51.3% vs 40.5% (p = 0.24)
**HIV/AIDS**							
Gardner 2005 USA [96]	316	Men or women with new diagnosis of HIV and not on antiretroviral therapy at an HIV clinic	In-person contact with Case Manager to build rapport, identify and address needs and barriers, provide encouragement, accompaniment if needed	Usual care—information pamphlet and referral to HIV provider	3 months (12 months)	**Attendance at HIV care provider at least twice in 12 months** Cost of intervention; HIV RNA viral load	**64% vs 49%, RRadj 1.41 (p = 0.006)**
Wohl 2006 USA [97]	250	Men or women with HIV and no more than one prior treatment regimen failure at public HIV clinics	Weekly meetings with Adherence Case Manager, addressed barriers, assistance with health insurance/finances, emotional and social support, treatment, legal services, nutritional support; 2nd intervention arm with directly observed medication therapy	Usual care including access to case manager and community supports; 2 of 3 clinics implemented adherence counselling outside of study	6 months (36 months)	**Viral load <400 copies/mL at 6 months** Log change in viral load; Change in CD4 cell count; Median CD4 cell count; Medication adherence; New or recurrent opportunistic infection	60% vs 54% (p = NS)
Wohl 2011 USA [98]	89	Inmates with HIV/AIDS approaching release seen at a state prison infectious disease clinic	Meetings before and after release with Case Manager, addressed patient needs including housing, employment, medical and social, assistance with patient goals, transfer to community services	Usual care + discharge planning and referrals	9 months	**Attendance at HIV clinic (at least one visit within 4, 12, 24 and 48 weeks)** Mean number of medical visits; Acute care utilization; Social service use; Re-incarceration	4 weeks: 65.1% vs 54.4% (p = 0.3); 12 weeks: 88.4% vs 78.3% (p = 0.2); 24 weeks: 90.7% vs 89.1% (P >0.5)
Metsch 2015 USA [99]	594	Low income men or women with HIV due for oral health services at HIV clinics	Case Manager provided in-person education, identified and addressed barriers, appointment scheduling, paperwork and transportation, referral to community services	Usual care	3 months (18 months)	**Attendance at dental care within 6 months**	**48.2% vs 25.7%, OR 2.52 (1.56, 4.08)**
Metsch 2016 USA [100]	801	Inpatient men or women with high risk HIV and concurrent substance use	Inpatient visit to encourage motivation and engagement, care coordination, review health information, address barriers and provide psychosocial support; study also included a group that received financial incentives	Usual care	6 months (12 months)	**Viral load < = 200 copies/mL at 12 months; death** HIV viral suppression at 6 months; Outpatient care with an HIV specialist; Presciption for HIV medications; HIV medication adherence; Substance use–related outcomes	37.5% vs 34.1%, not SS; 12.9% vs 11.7%, not SS
Giordano 2016 USA [101]	460	Inpatient men or women with HIV not engaged in HIV care	Inpatient visits followed by telephone contact to encourage active self-management, education, system navigation, address barriers, action planning and sharing stories	In person and telephone-based didactic education sessions focused on safer sex and drug use	10 weeks (6 months)	**Attendance at HIV clinic within 30 days AND between 31 and 180 days AND viral load < 400 copies/mL or decrease by 1 log at 6 months, if eligible for ART** Health-related QOL; Acute care utlization; ART adherence; viral load improvement; retention in care	28% vs 28% (p = 0.94)
Bassett 2016 South Africa [102]	523	Men or women that speak English or Zulu with HIV seen in primary care	In person meeting to provide support, education and address barriers, followed by series of phone calls to provide social support and address barriers and SMS reminders for tests and appointments	Usual care	4 months (9 months)	**Adherence to ART and/or TB treatment at 9 months if eligible for treatment** Mortality at 9 months	39% vs 42%; RR 0.93 (0.80, 1.08)
**Chronic kidney disease**							
Sullivan 2012 USA [103]	167	Men or women on dialysis and eligible for kidney transplant at community hemodialysis centers	Monthly meetings with Navigator to assess stage in transplant process and provide relevant assistance tailored to stage—education, motivation, facilitated referrals and communication, logistical support	Usual care	24 months	**Number of steps completed (up to 7) in transplant process**	**3.5 vs 1.6, difference 1.9 steps (1.3, 2.5)**
Navaneethan 2017 USA [104]	209	Adults ages 18–80 years old with eGFR 15–45 ml/min/1.73 m2 seen at family health centers	Monthly or quarterly in person meetings with intervening phone calls to address barriers, provide education, facilitate appointments, provide support and accompaniment if needed; study also included enhanced personal health record group	Usual care—advised to use their personal health record	24 months	**Change in eGFR over 2 years** Acquisition of laboratory measures; Prescription for renoprotective medications; Specialist referral rates; BP control; Hospitalizations and ED visits; Death	MD 0.4 ml/min/1.73m2 (-2.2, 3.1), not SS
**Dementia**							
Amjad 2017 USA [105]	303	Men and women with dementia and a caregiver in the community	In-person assessment and monthly contact for individualized care planning based on unmet needs and priorities, education and skill-building, referrals to services, counselling, and care monitoring	Augmented usual care—needs assessment, resource guide	18 months	**Health service utilization—acute, outpatient, and home/community**	No SS difference in any type
**Multiple chronic diseases**							
Kneipp 2011 USA [106]	432	Women receiving social assistance with at least one chronic health condition in an urban and rural welfare transition program	Meetings with Public Health Nurse Case Manager to initiate linkage with primary care, education, referrals, screening and routine care, and assisting with patient goals, Medicaid training session	Usual care	9 months	**Depression; General health; Functional status** Attendance at primary care and mental health visit; Knowledge of Medicaid benefits	**Depressive symptoms decreased more rapidly**; No SS difference for general health or functional status

### Chronic diseases

Patient navigation has been tested through RCTs more commonly in the context of cancer care (66%; n = 44) than in any other chronic disease. Of the cancer care studies, the majority were in cancer screening where the patient navigator’s focus was on helping the patient complete the screening test. Other chronic diseases where patient navigators have been studied include diabetes (n = 8), HIV (n = 7), cardiovascular disease (n = 4), chronic kidney disease (n = 2), dementia (n = 1) and multiple chronic diseases (n = 1).

### Intervention characteristics

Most navigator programs (64%) employed lay persons trained for the role. The primary mode of communication was by phone (90%) and over half were based in primary care or the community (57%). Patient navigators were responsible for a wide variety of activities. The most frequent strategy used by patient navigators to address health system barriers was care facilitation (i.e., making referrals, communicating with providers, coordinating care), followed by appointment scheduling (S2 Table). The most common activities used to address patient barriers included addressing patient attitudes and beliefs, appointment reminders, health literacy support and practical assistance (e.g., assistance with transportation, coordination of dependent care, arrangements for financial help or insurance benefits). Patient navigators most often provided education about the tests and treatments required in the form of discussion with patients. Many patient navigators also provided some form of direct psychosocial support to their patients.

Many studies (n = 26) reported using patient navigators that were culturally aligned, that is, a patient navigator who identified with the patient population in terms of ethnicity or other cultural factors, or included educational materials or communication approaches that were culturally tailored. Frequency of contact between navigators and patients ranged widely from only one contact to ‘as needed’ during the study duration, and duration of navigation varied widely.

### Outcomes

We found significant heterogeneity in primary outcomes. With respect to patient-oriented outcomes, one study included death as a primary outcome [100], two hypoglycaemia [84, 107], and five studies assessed quality of life and/or health status as a primary outcome: two in patients undergoing cancer treatment [41, 108], one in caregivers supporting patients undergoing cancer treatment[109], one in stroke[94], and one in patients with multiple chronic diseases[110]. Surrogate outcomes were most often reported in diabetes, where change in A1C levels was reported in seven studies [84, 86–88, 107, 111, 112]; three studies in HIV reported viral load [100, 101, 113] and one study in CKD reported change in estimated glomerular filtration rate (eGFR) [104]. Process outcomes were the most frequently reported primary outcome (n = 50), and they included completion of disease screening and adherence to follow-up procedures. Patient satisfaction or experience was reported as a primary outcome in one study[94], while hospitalizations and emergency room visits were reported as primary outcomes in three studies [84, 105, 107]. Of the 67 unique studies identified in this review, 45 (67%) reported a statistically significant improvement in one or more primary outcomes. We did not find an association between any program characteristics and the finding of a statistically significant improvement in a primary outcome.

Secondary outcomes were broader in scope, although many were variations of the primary outcome: for example, diagnostic resolution within a specified time period (where the primary outcome was time to diagnostic resolution). Secondary outcomes more frequently included patient-reported outcomes, including physical and mental health status, quality of life, and psychological distress. Other patient-oriented outcomes were reported as secondary outcomes: diagnostic outcomes of cancer screening and follow-up were reported in three studies[114–116], use of acute care was reported in four studies [101, 104, 109, 117], mortality in two [102, 104] and rate of opportunistic infections was reported in one study[113] Costs were considered in six studies [71, 73, 116, 118–120].

**Fig 3:**
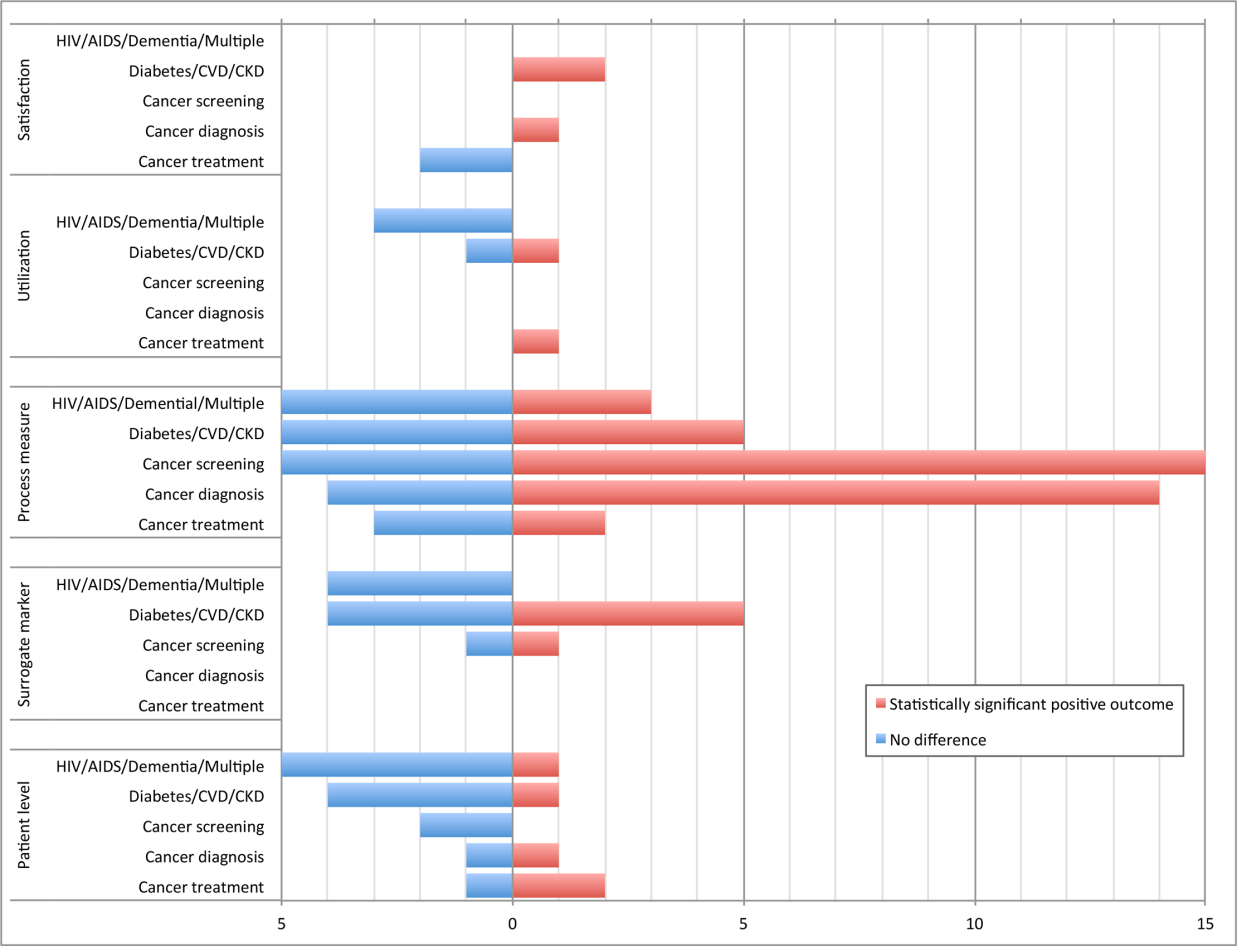
Number of studies reporting statistically significant positive vs null outcomes (primary or secondary) by outcome category.

Fig 3 depicts the number of studies that included outcomes within each category of interest (either as primary or secondary) and the proportion of these that demonstrated a statistically significant improvement. Studies were more likely to report positive results for process measures, and less so for surrogate markers, health care utilization, or patient-oriented outcomes. No studies found a negative impact from the patient navigator intervention.

With respect to publication bias, the proportion of studies reporting a statistically significant improvement in a primary outcome, across sample size quintiles, was 57%, 56%, 68%, 83%, and 69%.

## Discussion

Healthcare systems worldwide are under tremendous strain to address the needs of patients with chronic diseases. Since improving access to care and adherence to recommended treatment may improve outcomes, there is great interest in navigator programs. Our review of patient navigators for people with chronic diseases identified 54 unique randomized controlled studies. While most studies reported a positive effect of patient navigator programs for their primary outcome, the impact on clinical outcomes remains uncertain. The majority of the outcomes measured in RCTs of patient navigator studies reflect the process of receiving care; few studies assessed patient-oriented outcomes and many studies had short duration of follow-up, with uncertain power to detect an effect on clinical outcomes.

The variability of patient navigation programs found in the existing literature makes it challenging to make definitive statements about their effectiveness. We propose a consistent definition for a patient navigator, i.e., a person with or without a healthcare-related background that engages with patients on an individual basis to determine barriers to accessing care or following recommended guidelines, and provides information relevant to patients’ specific circumstances to facilitate self-management and access to care. The primary focus is on overcoming barriers, not providing clinical care, and in doing so patient navigators are often a source of social support.

Most other reviews of patient navigation have been restricted to cancer. The reviews by Wells and Paskett identified 33 studies of patient navigator programs in cancer, not restricted to RCTs [16, 28]. Even when examining patient navigation within one chronic disease (i.e., cancer), there was wide variation in intervention design, study design and study quality. In our review, a higher proportion of studies in cancer prevention or management (n = 32/44, 73%) reported a positive statistically significant effect for one or more primary outcomes versus studies in other chronic diseases (n = 13/23, 56%). Ali-Faisal et al published a systematic review and meta-analysis summarizing the effect of patient navigation programs on health care utilization, including adherence to screening and follow-up care, outcomes that our review classified as process outcomes[121]. Programs were required to use the term navigation or a variant in their description to be included in the review. The authors found that patient navigation was effective in increasing screening rates and improving adherence to recommended care; however, the effect on other health outcomes was less convincing and they noted considerable heterogeneity across studies. Our review of patient navigator programs expanded across chronic diseases and despite similar heterogeneity, echoed many of these previous findings and identified similar limitations.

Our study had strengths and limitations. Strengths include the thoroughness of our literature search, and our consideration of a broad group of diseases. A quantitative synthesis may have helped identify factors associated with successful patient navigator interventions, however this was precluded by the heterogeneity in both the intervention design and the outcomes reported. This heterogeneity also made it difficult to make definitive statements about the merit of specific patient navigator activities. We were unable to identify the most important elements of patient navigator programs that were associated with an improvement in the primary outcome. Other potential reasons include incomplete reporting (i.e., some program elements may have been present but not reported) or variation in how the individual features were implemented within programs. Though we were unable to summarize our results quantitatively, a descriptive review of these randomized controlled trials provides a comprehensive summary of navigator programs and outcomes reported. Our review was limited to published reports of randomized controlled trials, and therefore, although we noted a trend toward a lower proportion of small studies reporting statistically significant positive results, we could not rule out publication bias.

## Conclusions

Our findings indicate that patient navigator programs improve processes of care, although few studies assessed patient experience, clinical outcomes or costs. The inability to definitively outline successful components remains a key uncertainty in the use of patient navigator programs across chronic diseases. Given the increasing popularity of patient navigator interventions, future studies should use consistent definitions for patient navigator interventions, and in addition to determining which elements of the intervention are most likely to lead to improved outcomes, studies should focus on patient experience and disease-specific clinical outcomes that are important to patients.

## Supporting information

S1 File MEDLINE search strategy. (PDF)

S2 File PRISMA checklist. (PDF)

S1 Table Risk of bias by individual study. (PDF)

S2 Table Attributes of patient navigator interventions. (PDF)

## References

[1] Ferlay J, Shin HR, Bray F, Forman D, Mathers C, Parkin DM. Estimates of worldwide burden of cancer in 2008: GLOBOCAN 2008. Int J Cancer. 2010;127(12):2893–917. doi: 10.1002/ijc.25516 . 21351269 10.1002/ijc.25516

[2] Shaw JE, Sicree RA, Zimmet PZ. Global estimates of the prevalence of diabetes for 2010 and 2030. Diabetes Res Clin Pract. 2010;87(1):4–14. doi: 10.1016/j.diabres.2009.10.007 . 19896746 10.1016/j.diabres.2009.10.007

[3] How Do Sicker Canadians with Chronic Disease Rate the Health Care System? Results from the 2011 Commonwealth Fund International Health Policy Survey of Sicker Adults. Toronto: Health Council of Canada, 2011.

[4] Broemeling AM, Watson DE, Prebtani F. Population patterns of chronic health conditions, co-morbidity and healthcare use in Canada: implications for policy and practice. Healthcare quarterly (Toronto, Ont). 2008;11(3):70–6. Epub 2008/06/10. . 18536538 10.12927/hcq.2008.19859

[5] Cost effectiveness analysis of improved blood pressure control in hypertensive patients with type 2 diabetes: UKPDS 40. UK Prospective Diabetes Study Group. BMJ. 1998;317(7160):720–6. Epub 1998/09/11. . 9732339 PMC28661

[6] Cost-effectiveness of intensive glycemic control, intensified hypertension control, and serum cholesterol level reduction for type 2 diabetes. JAMA. 2002;287(19):2542–51. Epub 2002/05/22. doi: joc20021 [pii]. . 12020335 10.1001/jama.287.19.2542

[7] Gray A, Raikou M, McGuire A, Fenn P, Stevens R, Cull C, et al Cost effectiveness of an intensive blood glucose control policy in patients with type 2 diabetes: economic analysis alongside randomised controlled trial (UKPDS 41). United Kingdom Prospective Diabetes Study Group. BMJ. 2000;320(7246):1373–8. Epub 2000/05/20. . 10.1136/bmj.320.7246.1373 PMC27380 10818026

[8] Manns BJ, Tonelli M, Zhang J, Campbell DJ, Sargious P, Ayyalasomayajula B, et al Enrolment in primary care networks: impact on outcomes and processes of care for patients with diabetes. CMAJ. 2012;184(2):E144–52. Epub 2011/12/07. doi: 10.1503/cmaj.110755 ; PubMed Central PMCID: PMC3273535. 22143232 10.1503/cmaj.110755 PMC3273535

[9] McAlister FA, Majumdar SR, Eurich DT, Johnson JA. The effect of specialist care within the first year on subsequent outcomes in 24,232 adults with new-onset diabetes mellitus: population-based cohort study. Qual Saf Health Care. 2007;16(1):6–11. Epub 2007/02/16. doi: 10.1136/qshc.2006.018648 ; PubMed Central PMCID: PMC2464930. 17301194 10.1136/qshc.2006.018648 PMC2464930

[10] Shah BR, Hux JE, Austin PC. Diabetes is not treated as a coronary artery disease risk equivalent. Diabetes Care. 2007;30(2):381–3. Epub 2007/01/30. doi: 10.2337/dc06-1654 . 17259516 10.2337/dc06-1654

[11] Toth EL, Majumdar SR, Guirguis LM, Lewanczuk RZ, Lee TK, Johnson JA. Compliance with clinical practice guidelines for type 2 diabetes in rural patients: treatment gaps and opportunities for improvement. Pharmacotherapy. 2003;23(5):659–65. Epub 2003/05/14. . 12741441 10.1592/phco.23.5.659.32203

[12] Ferlie EB, Shortell SM. Improving the quality of health care in the United Kingdom and the United States: a framework for change. Milbank Q. 2001;79(2):281–315. Epub 2001/07/07. doi: 10.1111/1468-0009.00206 . 11439467 10.1111/1468-0009.00206 PMC2751188

[13] Genoff MC, Zaballa A, Gany F, Gonzalez J, Ramirez J, Jewell ST, et al Navigating Language Barriers: A Systematic Review of Patient Navigators' Impact on Cancer Screening for Limited English Proficient Patients. J Gen Intern Med. 2016;31(4):426–34. doi: 10.1007/s11606-015-3572-3 ; PubMed Central PMCID: PMCPMC4803699. 26786875 10.1007/s11606-015-3572-3 PMC4803699

[14] Parker VA, Lemak CH. Navigating patient navigation: crossing health services research and clinical boundaries. Advances in health care management. 2011;11:149–83. Epub 2011/01/01. . 22908669 10.1108/s1474-8231(2011)0000011010

[15] Pedersen A, Hack TF. Pilots of oncology health care: a concept analysis of the patient navigator role. Oncol Nurs Forum. 2010;37(1):55–60. doi: 10.1188/10.ONF.55-60 20044339 10.1188/10.ONF.55-60

[16] Wells KJ, Battaglia TA, Dudley DJ, Garcia R, Greene A, Calhoun E, et al Patient navigation: state of the art or is it science? Cancer. 2008;113(8):1999–2010. Epub 2008/09/10. doi: 10.1002/cncr.23815 ; PubMed Central PMCID: PMCPMC2679696. 18780320 10.1002/cncr.23815 PMC2679696

[17] Freeman HP. The history, principles, and future of patient navigation: commentary. Seminars in oncology nursing. 2013;29(2):72–5. Epub 2013/05/09. doi: 10.1016/j.soncn.2013.02.002 . 23651676 10.1016/j.soncn.2013.02.002

[18] Walkinshaw E. Patient navigators becoming the norm in Canada. CMAJ. 2011;183(15):E1109–10. Epub 2011/09/21. doi: 10.1503/cmaj.109-3974 ; PubMed Central PMCID: PMCPMC3193138. 21930738 10.1503/cmaj.109-3974 PMC3193138

[19] Thom DH, Ghorob A, Hessler D, De Vore D, Chen E, Bodenheimer TA. Impact of peer health coaching on glycemic control in low-income patients with diabetes: a randomized controlled trial. Ann Fam Med. 2013;11(2):137–44. doi: 10.1370/afm.1443 ; PubMed Central PMCID: PMCPMC3601392. 23508600 10.1370/afm.1443 PMC3601392

[20] Lasser KE, Kenst KS, Quintiliani LM, Wiener RS, Murillo J, Pbert L, et al Patient navigation to promote smoking cessation among low-income primary care patients: a pilot randomized controlled trial. J Ethn Subst Abuse. 2013;12(4):374–90. doi: 10.1080/15332640.2013.819311 ; PubMed Central PMCID: PMCPMC3827692. 24215228 10.1080/15332640.2013.819311 PMC3827692

[21] Quintiliani LM, Russinova ZL, Bloch PP, Truong V, Xuan Z, Pbert L, et al Patient navigation and financial incentives to promote smoking cessation in an underserved primary care population: A randomized controlled trial protocol. Contemp Clin Trials. 2015;45(Pt B):449–57. doi: 10.1016/j.cct.2015.09.005 . 26362691 10.1016/j.cct.2015.09.005

[22] Enard KR, Nevarez L, Hernandez M, Hovick SR, Moguel MR, Hajek RA, et al Patient navigation to increase colorectal cancer screening among Latino Medicare enrollees: a randomized controlled trial. Cancer Causes Control. 2015;26(9):1351–9. doi: 10.1007/s10552-015-0620-6 . 26109462 10.1007/s10552-015-0620-6 PMC5215648

[23] Fischer SM, Sauaia A, Kutner JS. Patient navigation: a culturally competent strategy to address disparities in palliative care. Journal of palliative medicine. 2007;10(5):1023–8. Epub 2007/11/08. doi: 10.1089/jpm.2007.0070 . 17985954 10.1089/jpm.2007.0070

[24] Shlay JC, Barber B, Mickiewicz T, Maravi M, Drisko J, Estacio R, et al Reducing cardiovascular disease risk using patient navigators, Denver, Colorado, 2007–2009. Preventing chronic disease. 2011;8(6):A143 Epub 2011/10/19. ; PubMed Central PMCID: PMCPMC3221582. 22005636 PMC3221582

[25] Goff SL, Pekow PS, White KO, Lagu T, Mazor KM, Lindenauer PK. IDEAS for a healthy baby—reducing disparities in use of publicly reported quality data: study protocol for a randomized controlled trial. Trials. 2013;14:244 Epub 2013/08/08. doi: 10.1186/1745-6215-14-244 ; PubMed Central PMCID: PMCPMC3751013. 23919671 10.1186/1745-6215-14-244 PMC3751013

[26] Scott LB, Gravely S, Sexton TR, Brzostek S, Brown DL. Examining the Effect of a Patient Navigation Intervention on Outpatient Cardiac Rehabilitation Awareness and Enrollment. Journal of cardiopulmonary rehabilitation and prevention. 2013;33(5):281–91. Epub 2013/07/05. doi: 10.1097/HCR.0b013e3182972dd6 ; PubMed Central PMCID: PMCPMC3759655. 23823904 10.1097/HCR.0b013e3182972dd6 PMC3759655

[27] Darnell JS. Navigators and assisters: two case management roles for social workers in the Affordable Care Act. Health & social work. 2013;38(2):123–6. Epub 2013/07/20. . 23865289 10.1093/hsw/hlt003

[28] Paskett ED, Harrop JP, Wells KJ. Patient navigation: an update on the state of the science. CA: a cancer journal for clinicians. 2011;61(4):237–49. Epub 2011/06/11. doi: 10.3322/caac.20111 ; PubMed Central PMCID: PMCPMC3623288. 21659419 10.3322/caac.20111 PMC3623288

[29] Monza K, Harris D, Shaw C. The Role of the Nurse Navigator in the Management of the Heart Failure Patient. Critical care nursing clinics of North America. 2015;27(4):537–49. Epub 2015/11/17. doi: 10.1016/j.cnc.2015.07.010 . 26567497 10.1016/j.cnc.2015.07.010

[30] Loskutova NY, Tsai AG, Fisher EB, LaCruz DM, Cherrington AL, Harrington TM, et al Patient Navigators Connecting Patients to Community Resources to Improve Diabetes Outcomes. Journal of the American Board of Family Medicine: JABFM. 2016;29(1):78–89. Epub 2016/01/16. doi: 10.3122/jabfm.2016.01.150048 . 26769880 10.3122/jabfm.2016.01.150048

[31] Gardner E. A Personal Compass: What Patient Navigation Can Do for You. US News 2015 9 16, 2015.

[32] PRESS Peer Review of Electronic Search Strategies: 2015 Guideline Explanation and Elaboration (PRESS E&E). Ottawa: 2016 January 2016. Report No.

[33] Canadian Community Health Survey 2012: Statistics Canada; 2012 [January 15, 2013]. Available from: http://www23.statcan.gc.ca/imdb/p3Instr.pl?Function=getInstrumentList&Item_Id=119788&UL=1V&.

[34] Dohan D, Schrag D. Using navigators to improve care of underserved patients: current practices and approaches. Cancer. 2005;104(4):848–55. doi: 10.1002/cncr.21214 . 16010658 10.1002/cncr.21214

[35] Kelly E, Ivers N, Zawi R, Barnieh L, Manns B, Lorenzetti DL, et al Patient navigators for people with chronic disease: protocol for a systematic review and meta-analysis. Systematic Reviews. 2015;4 doi: 10.1186/s13643-015-0019-1 . 25874724 10.1186/s13643-015-0019-1 PMC4375835

[36] Fiscella K, Whitley E, Hendren S, Raich P, Humiston S, Winters P, et al Patient navigation for breast and colorectal cancer treatment: a randomized trial. Cancer epidemiology, biomarkers & prevention: a publication of the American Association for Cancer Research, cosponsored by the American Society of Preventive Oncology. 2012;21(10):1673–81. Epub 2012/10/10. doi: 10.1158/1055-9965.epi-12-0506 ; PubMed Central PMCID: PMCPMC3724524. 23045542 10.1158/1055-9965.EPI-12-0506 PMC3724524

[37] Hendren S, Griggs JJ, Epstein R, Humiston S, Jean-Pierre P, Winters P, et al Randomized controlled trial of patient navigation for newly diagnosed cancer patients: effects on quality of life. Cancer epidemiology, biomarkers & prevention: a publication of the American Association for Cancer Research, cosponsored by the American Society of Preventive Oncology. 2012;21(10):1682–90. Epub 2012/10/10. doi: 10.1158/1055-9965.epi-12-0537 ; PubMed Central PMCID: PMCPMC3468902. 23045543 10.1158/1055-9965.EPI-12-0537 PMC3468902

[38] Ell K, Vourlekis B, Xie B, Nedjat-Haiem FR, Lee PJ, Muderspach L, et al Cancer treatment adherence among low-income women with breast or gynecologic cancer: a randomized controlled trial of patient navigation. Cancer. 2009;115(19):4606–15. Epub 2009/06/25. doi: 10.1002/cncr.24500 ; PubMed Central PMCID: PMCPMC2749894. 19551881 10.1002/cncr.24500 PMC2749894

[39] White VM, Macvean ML, Grogan S, D'Este C, Akkerman D, Ieropoli S, et al Can a tailored telephone intervention delivered by volunteers reduce the supportive care needs, anxiety and depression of people with colorectal cancer? A randomised controlled trial. Psycho-oncology. 2012;21(10):1053–62. Epub 2011/07/20. doi: 10.1002/pon.2019 . 21769989 10.1002/pon.2019

[40] Percac-Lima S, Cronin PR, Ryan DP, Chabner BA, Daly EA, Kimball AB. Patient navigation based on predictive modeling decreases no-show rates in cancer care. Cancer. 2015;121(10):1662–70. Epub 2015/01/15. doi: 10.1002/cncr.29236 . 25585595 10.1002/cncr.29236

[41] Giese-Davis J, Bliss-Isberg C, Wittenberg L, White J, Star P, Zhong L, et al Peer-counseling for women newly diagnosed with breast cancer: A randomized community/research collaboration trial. Cancer. 2016;122(15):2408–17. doi: 10.1002/cncr.30036 . 27198057 10.1002/cncr.30036

[42] Shaw Joanne, Young Jane, Butow Phyllis, et al Improving psychosocial outcomes for caregivers of people with poor prognosis gastrointestinal cancers: a randomized controlled trial (Family Connect). Supportive Care in Cancer. 2016;24(2):585–95. doi: 10.1007/s00520-015-2817-3 . Language: English. Entry Date: 20160804. Revision Date: 20170601. Publication Type: journal article. Journal Subset: Biomedical. 26111955 10.1007/s00520-015-2817-3

[43] Ferrante JM, Chen PH, Kim S. The effect of patient navigation on time to diagnosis, anxiety, and satisfaction in urban minority women with abnormal mammograms: a randomized controlled trial. Journal of urban health: bulletin of the New York Academy of Medicine. 2008;85(1):114–24. Epub 2007/10/02. doi: 10.1007/s11524-007-9228-9 ; PubMed Central PMCID: PMCPMC2430139. 17906931 10.1007/s11524-007-9228-9 PMC2430139

[44] Ell K, Vourlekis B, Lee PJ, Xie B. Patient navigation and case management following an abnormal mammogram: a randomized clinical trial. Preventive medicine. 2007;44(1):26–33. Epub 2006/09/12. doi: 10.1016/j.ypmed.2006.08.001 . 16962652 10.1016/j.ypmed.2006.08.001

[45] Crump SR, Shipp MP, McCray GG, Morris SJ, Okoli JA, Caplan LS, et al Abnormal mammogram follow-up: do community lay health advocates make a difference? Health promotion practice. 2008;9(2):140–8. Epub 2008/03/15. doi: 10.1177/1524839907312806 . 18340089 10.1177/1524839907312806

[46] Maxwell AE, Jo AM, Crespi CM, Sudan M, Bastani R. Peer navigation improves diagnostic follow-up after breast cancer screening among Korean American women: results of a randomized trial. Cancer Causes Control. 2010;21(11):1931–40. Epub 2010/08/03. doi: 10.1007/s10552-010-9621-7 ; PubMed Central PMCID: PMCPMC2959157. 20676928 10.1007/s10552-010-9621-7 PMC2959157

[47] Bastani R, Mojica CM, Berman BA, Ganz PA. Low-income women with abnormal breast findings: results of a randomized trial to increase rates of diagnostic resolution. Cancer epidemiology, biomarkers & prevention: a publication of the American Association for Cancer Research, cosponsored by the American Society of Preventive Oncology. 2010;19(8):1927–36. Epub 2010/07/22. doi: 10.1158/1055-9965.epi-09-0481 . 20647406 10.1158/1055-9965.EPI-09-0481

[48] Lerman C, Hanjani P, Caputo C, Miller S, Delmoor E, Nolte S, et al Telephone counseling improves adherence to colposcopy among lower-income minority women. Journal of clinical oncology: official journal of the American Society of Clinical Oncology. 1992;10(2):330–3. Epub 1992/02/01. doi: 10.1200/JCO.1992.10.2.330 . 1732434 10.1200/JCO.1992.10.2.330

[49] Miller SM, Siejak KK, Schroeder CM, Lerman C, Hernandez E, Helm CW. Enhancing adherence following abnormal Pap smears among low-income minority women: a preventive telephone counseling strategy. Journal of the National Cancer Institute. 1997;89(10):703–8. Epub 1997/05/21. . 9168185 10.1093/jnci/89.10.703

[50] Engelstad LP, Stewart S, Otero-Sabogal R, Leung MS, Davis PI, Pasick RJ. The effectiveness of a community outreach intervention to improve follow-up among underserved women at highest risk for cervical cancer. Preventive medicine. 2005;41(3–4):741–8. Epub 2005/08/30. doi: 10.1016/j.ypmed.2005.06.003 . 16125761 10.1016/j.ypmed.2005.06.003

[51] Lee JH, Fulp W, Wells KJ, Meade CD, Calcano E, Roetzheim R. Patient navigation and time to diagnostic resolution: results for a cluster randomized trial evaluating the efficacy of patient navigation among patients with breast cancer screening abnormalities, Tampa, FL. PloS one. 2013;8(9):e74542 Epub 2013/09/26. doi: 10.1371/journal.pone.0074542 ; PubMed Central PMCID: PMCPMC3774725. 24066145 10.1371/journal.pone.0074542 PMC3774725

[52] Lee JH, Fulp W, Wells KJ, Meade CD, Calcano E, Roetzheim R. Effect of patient navigation on time to diagnostic resolution among patients with colorectal cancer-related abnormalities. Journal of cancer education: the official journal of the American Association for Cancer Education. 2014;29(1):144–50. Epub 2013/10/12. doi: 10.1007/s13187-013-0561-2 ; PubMed Central PMCID: PMCPMC3945676. 24113902 10.1007/s13187-013-0561-2 PMC3945676

[53] Wells KJ, Lee JH, Calcano ER, Meade CD, Rivera M, Fulp WJ, et al A cluster randomized trial evaluating the efficacy of patient navigation in improving quality of diagnostic care for patients with breast or colorectal cancer abnormalities. Cancer epidemiology, biomarkers & prevention: a publication of the American Association for Cancer Research, cosponsored by the American Society of Preventive Oncology. 2012;21(10):1664–72. Epub 2012/10/10. doi: 10.1158/1055-9965.epi-12-0448 ; PubMed Central PMCID: PMCPMC3511588. 23045541 10.1158/1055-9965.EPI-12-0448 PMC3511588

[54] Raich PC, Whitley EM, Thorland W, Valverde P, Fairclough D. Patient navigation improves cancer diagnostic resolution: an individually randomized clinical trial in an underserved population. Cancer epidemiology, biomarkers & prevention: a publication of the American Association for Cancer Research, cosponsored by the American Society of Preventive Oncology. 2012;21(10):1629–38. Epub 2012/10/10. doi: 10.1158/1055-9965.epi-12-0513 ; PubMed Central PMCID: PMCPMC4053249. 23045537 10.1158/1055-9965.EPI-12-0513 PMC4053249

[55] Paskett ED, Katz ML, Post DM, Pennell ML, Young GS, Seiber EE, et al The Ohio Patient Navigation Research Program: does the American Cancer Society patient navigation model improve time to resolution in patients with abnormal screening tests? Cancer epidemiology, biomarkers & prevention: a publication of the American Association for Cancer Research, cosponsored by the American Society of Preventive Oncology. 2012;21(10):1620–8. Epub 2012/10/10. doi: 10.1158/1055-9965.epi-12-0523 ; PubMed Central PMCID: PMCPMC3785236. 23045536 10.1158/1055-9965.EPI-12-0523 PMC3785236

[56] Weber BE, Reilly BM. Enhancing mammography use in the inner city. A randomized trial of intensive case management. Archives of internal medicine. 1997;157(20):2345–9. Epub 1997/11/15. . 9361575

[57] West DS, Greene P, Pulley L, Kratt P, Gore S, Weiss H, et al Stepped-care, community clinic interventions to promote mammography use among low-income rural African American women. Health education & behavior: the official publication of the Society for Public Health Education. 2004;31(4 Suppl):29S–44S. Epub 2004/08/07. doi: 10.1177/1090198104266033 . 15296690 10.1177/1090198104266033

[58] Paskett E, Tatum C, Rushing J, Michielutte R, Bell R, Long Foley K, et al Randomized trial of an intervention to improve mammography utilization among a triracial rural population of women. Journal of the National Cancer Institute. 2006;98(17):1226–37. Epub 2006/09/07. doi: 10.1093/jnci/djj333 ; PubMed Central PMCID: PMCPMC4450352. 16954475 10.1093/jnci/djj333 PMC4450352

[59] Rahm AK, Sukhanova A, Ellis J, Mouchawar J. Increasing utilization of cancer genetic counseling services using a patient navigator model. Journal of genetic counseling. 2007;16(2):171–7. Epub 2007/02/06. doi: 10.1007/s10897-006-9051-6 . 17277995 10.1007/s10897-006-9051-6

[60] Ahmed NU, Haber G, Semenya KA, Hargreaves MK. Randomized controlled trial of mammography intervention in insured very low-income women. Cancer epidemiology, biomarkers & prevention: a publication of the American Association for Cancer Research, cosponsored by the American Society of Preventive Oncology. 2010;19(7):1790–8. Epub 2010/07/01. doi: 10.1158/1055-9965.epi-10-0141 . 20587669 10.1158/1055-9965.EPI-10-0141

[61] Phillips CE, Rothstein JD, Beaver K, Sherman BJ, Freund KM, Battaglia TA. Patient navigation to increase mammography screening among inner city women. J Gen Intern Med. 2011;26(2):123–9. Epub 2010/10/12. doi: 10.1007/s11606-010-1527-2 ; PubMed Central PMCID: PMCPMC3019333. 20931294 10.1007/s11606-010-1527-2 PMC3019333

[62] Marshall JK, Mbah OM, Ford JG, Phelan-Emrick D, Ahmed S, Bone L, et al Effect of Patient Navigation on Breast Cancer Screening Among African American Medicare Beneficiaries: A Randomized Controlled Trial. J Gen Intern Med. 2016;31(1):68–76. Epub 2015/08/12. doi: 10.1007/s11606-015-3484-2 ; PubMed Central PMCID: PMCPMC4700012. 26259762 10.1007/s11606-015-3484-2 PMC4700012

[63] Taylor VM, Hislop TG, Jackson JC, Tu SP, Yasui Y, Schwartz SM, et al A randomized controlled trial of interventions to promote cervical cancer screening among Chinese women in North America. Journal of the National Cancer Institute. 2002;94(9):670–7. Epub 2002/05/02. ; PubMed Central PMCID: PMCPMC1592333. 11983755 10.1093/jnci/94.9.670 PMC1592333

[64] Taylor VM, Jackson JC, Yasui Y, Nguyen TT, Woodall E, Acorda E, et al Evaluation of a cervical cancer control intervention using lay health workers for Vietnamese American women. American journal of public health. 2010;100(10):1924–9. Epub 2010/08/21. doi: 10.2105/AJPH.2009.190348 ; PubMed Central PMCID: PMCPMC2936992. 20724673 10.2105/AJPH.2009.190348 PMC2936992

[65] Jandorf L, Gutierrez Y, Lopez J, Christie J, Itzkowitz SH. Use of a patient navigator to increase colorectal cancer screening in an urban neighborhood health clinic. Journal of urban health: bulletin of the New York Academy of Medicine. 2005;82(2):216–24. Epub 2005/05/13. doi: 10.1093/jurban/jti046 ; PubMed Central PMCID: PMCPMC3456577. 15888638 10.1093/jurban/jti046 PMC3456577

[66] Basch CE, Wolf RL, Brouse CH, Shmukler C, Neugut A, DeCarlo LT, et al Telephone outreach to increase colorectal cancer screening in an urban minority population. American journal of public health. 2006;96(12):2246–53. Epub 2006/11/02. doi: 10.2105/AJPH.2005.067223 ; PubMed Central PMCID: PMCPMC1698159. 17077394 10.2105/AJPH.2005.067223 PMC1698159

[67] Percac-Lima S, Grant RW, Green AR, Ashburner JM, Gamba G, Oo S, et al A culturally tailored navigator program for colorectal cancer screening in a community health center: a randomized, controlled trial. J Gen Intern Med. 2009;24(2):211–7. Epub 2008/12/11. doi: 10.1007/s11606-008-0864-x ; PubMed Central PMCID: PMCPMC2628981. 19067085 10.1007/s11606-008-0864-x PMC2628981

[68] Christie J, Itzkowitz S, Lihau-Nkanza I, Castillo A, Redd W, Jandorf L. A randomized controlled trial using patient navigation to increase colonoscopy screening among low-income minorities. Journal of the National Medical Association. 2008;100(3):278–84. Epub 2008/04/09. . 18390020 10.1016/s0027-9684(15)31240-2

[69] Lasser KE, Murillo J, Lisboa S, Casimir AN, Valley-Shah L, Emmons KM, et al Colorectal cancer screening among ethnically diverse, low-income patients: a randomized controlled trial. Archives of internal medicine. 2011;171(10):906–12. Epub 2011/05/25. doi: 10.1001/archinternmed.2011.201 . 21606094 10.1001/archinternmed.2011.201

[70] Coronado GD, Golovaty I, Longton G, Levy L, Jimenez R. Effectiveness of a clinic-based colorectal cancer screening promotion program for underserved Hispanics. Cancer. 2011;117(8):1745–54. Epub 2011/04/08. doi: 10.1002/cncr.25730 . 21472722 10.1002/cncr.25730

[71] Green BB, Wang CY, Anderson ML, Chubak J, Meenan RT, Vernon SW, et al An automated intervention with stepped increases in support to increase uptake of colorectal cancer screening: a randomized trial. Ann Intern Med. 2013;158(5 Pt 1):301–11. Epub 03/06. doi: 10.7326/0003-4819-158-5-201303050-00002 . 23460053 10.7326/0003-4819-158-5-201303050-00002 PMC3953144

[72] Lairson DR, Dicarlo M, Deshmuk AA, Fagan HB, Sifri R, Katurakes N, et al Cost-effectiveness of a standard intervention versus a navigated intervention on colorectal cancer screening use in primary care. Cancer. 2014;120(7):1042–9. Epub 2014/01/18. doi: 10.1002/cncr.28535 ; PubMed Central PMCID: PMCPMC3961516. 24435411 10.1002/cncr.28535 PMC3961516

[73] Myers RE, Bittner-Fagan H, Daskalakis C, Sifri R, Vernon SW, Cocroft J, et al A randomized controlled trial of a tailored navigation and a standard intervention in colorectal cancer screening. Cancer Epidemiol Biomarkers Prev. 2013;22(1):109–17. Epub 2012/11/03. doi: 10.1158/1055-9965.EPI-12-0701 PubMed Central PMCID: PMCPMC5537598. 23118143 10.1158/1055-9965.EPI-12-0701 PMC5537598

[74] Myers RE, Sifri R, Daskalakis C, DiCarlo M, Geethakumari PR, Cocroft J, et al Increasing colon cancer screening in primary care among African Americans. Journal of the National Cancer Institute. 2014;106(12). Epub 2014/12/08. doi: 10.1093/jncimonographs/lgu003 PubMed PMID: 25174031; PubMed Central PMCID: PMCPMC4817126. 25481829 10.1093/jnci/dju344 PMC4817126

[75] Ritvo PG, Myers RE, Paszat LF, Tinmouth JM, McColeman J, Mitchell B, et al Personal navigation increases colorectal cancer screening uptake. Cancer epidemiology, biomarkers & prevention: a publication of the American Association for Cancer Research, cosponsored by the American Society of Preventive Oncology. 2015;24(3):506–11. Epub 2014/11/08. doi: 10.1158/1055-9965.epi-14-0744 . 25378365 10.1158/1055-9965.EPI-14-0744

[76] Greenspan M, Shawron K, Avery E, Barnes LL, Olinger T, Li H, et al Patient directed navigation does not improve adherence to colonoscopy or bowel preparation quality: A randomized controlled trial. Gastroenterology. 2016;150(4 SUPPL. 1):S757. PubMed PMID: CN-01160715 NEW.

[77] Cole H, Thompson HS, White M, Browne R, Trinh-Shevrin C, Braithwaite S, et al Community-Based, Preclinical Patient Navigation for Colorectal Cancer Screening Among Older Black Men Recruited From Barbershops: The MISTER B Trial. American Journal of Public Health. 2017;107(9):1433–40. doi: 10.2105/AJPH.2017.303885 . 28727540 10.2105/AJPH.2017.303885 PMC5551599

[78] Guillaume E, Dejardin O, Bouvier V, et al Patient navigation to reduce social inequalities in colorectal cancer screening participation: A cluster randomized controlled trial. Preventive Medicine. 2017;16:16 doi: 10.1016/j.ypmed.2017.08.012 PubMed PMID: 28823681. 10.1016/j.ypmed.2017.08.012 28823681

[79] DeGroff A, Schroy P C, Morrissey rd, et al Patient Navigation for Colonoscopy Completion: Results of an RCT. American Journal of Preventive Medicine. 2017;53(3):363–72. doi: 10.1016/j.amepre.2017.05.010 . 28676254 10.1016/j.amepre.2017.05.010 PMC8855664

[80] Dietrich AJ, Tobin JN, Cassells A, Robinson CM, Greene MA, Sox CH, et al Telephone care management to improve cancer screening among low-income women: a randomized, controlled trial. Annals of internal medicine. 2006;144(8):563–71. Epub 2006/04/19. ; PubMed Central PMCID: PMCPMC3841972. 16618953 10.7326/0003-4819-144-8-200604180-00006 PMC3841972

[81] Braun KL, Thomas WL Jr., Domingo JL, Allison AL, Ponce A, Haunani Kamakana P, et al Reducing cancer screening disparities in medicare beneficiaries through cancer patient navigation. Journal of the American Geriatrics Society. 2015;63(2):365–70. Epub 2015/02/03. doi: 10.1111/jgs.13192 ; PubMed Central PMCID: PMCPMC4850231. 25640884 10.1111/jgs.13192 PMC4850231

[82] Percac-Lima S, Ashburner JM, Zai AH, Chang Y, Oo SA, Guimaraes E, et al Patient Navigation for Comprehensive Cancer Screening in High-Risk Patients Using a Population-Based Health Information Technology System: A Randomized Clinical Trial. JAMA Internal Medicine. 2016;176(7):930–7. doi: 10.1001/jamainternmed.2016.0841 . 27273602 10.1001/jamainternmed.2016.0841

[83] Corkery E, Palmer C, Foley ME, Schechter CB, Frisher L, Roman SH. Effect of a bicultural community health worker on completion of diabetes education in a Hispanic population. Diabetes Care. 1997;20(3):254–7. Epub 1997/03/01. . 9051367 10.2337/diacare.20.3.254

[84] Laffel LM, Brackett J, Ho J, Anderson BJ. Changing the process of diabetes care improves metabolic outcomes and reduces hospitalizations. Qual Manag Health Care. 1998;6(4):53–62. Epub 05/29. . 10339045 10.1097/00019514-199806040-00006

[85] Svoren BM, Butler D, Levine BS, Anderson BJ, Laffel LM. Reducing acute adverse outcomes in youths with type 1 diabetes: a randomized, controlled trial. Pediatrics. 2003;112(4):914–22. Epub 2003/10/03. . 14523186 10.1542/peds.112.4.914

[86] Gary TL, Batts-Turner M, Bone LR, Yeh HC, Wang NY, Hill-Briggs F, et al A randomized controlled trial of the effects of nurse case manager and community health worker team interventions in urban African-Americans with type 2 diabetes. Control Clin Trials. 2004;25(1):53–66. Epub 2004/02/26. doi: 10.1016/j.cct.2003.10.010 . 14980748 10.1016/j.cct.2003.10.010

[87] Spencer MS, Rosland AM, Kieffer EC, Sinco BR, Valerio M, Palmisano G, et al Effectiveness of a community health worker intervention among African American and Latino adults with type 2 diabetes: a randomized controlled trial. American journal of public health. 2011;101(12):2253–60. Epub 2011/06/18. doi: 10.2105/AJPH.2010.300106 ; PubMed Central PMCID: PMCPMC3222418. 21680932 10.2105/AJPH.2010.300106 PMC3222418

[88] Prezio EA, Cheng D, Balasubramanian BA, Shuval K, Kendzor DE, Culica D. Community Diabetes Education (CoDE) for uninsured Mexican Americans: A randomized controlled trial of a culturally tailored diabetes education and management program led by a community health worker. Diabetes Res Clin Pract. 2013;100(1):19–28. doi: 10.1016/j.diabres.2013.01.027 . Language: English. Entry Date: In Process. Revision Date: 20130503. Publication Type: journal article. Journal Subset: Biomedical. 23453178 10.1016/j.diabres.2013.01.027

[89] Carrasquillo O, Alonzo Y, Lebron C, Ferras N, Reyes-Arrechea E, Kenya S. A randomized trial of a community health worker led intervention to improve diabetes intermediate outcomes among latino patients with poorly controlled diabetes. Journal of General Internal Medicine. 2014;29:S13.

[90] Thom DH, Hessler D, Willard-Grace R, Bodenheimer T, Najmabadi A, Araujo C, et al Does health coaching change patients' trust in their primary care provider? Patient education and counseling. 2014;96(1):135–8. Epub 2014/04/30. doi: 10.1016/j.pec.2014.03.018 . 24776175 10.1016/j.pec.2014.03.018

[91] Thom DH, Hessler D, Willard-Grace R, DeVore D, Prado C, Bodenheimer T, et al Health coaching by medical assistants improves patients' chronic care experience. The American journal of managed care. 2015;21(10):685–91. Epub 2015/12/04. . 26633093

[92] Willard-Grace R, Chen EH, Hessler D, DeVore D, Prado C, Bodenheimer T, et al Health coaching by medical assistants to improve control of diabetes, hypertension, and hyperlipidemia in low-income patients: a randomized controlled trial. Ann Fam Med. 2015;13(2):130–8. Epub 2015/03/11. doi: 10.1370/afm.1768 ; PubMed Central PMCID: PMCPMC4369595. 25755034 10.1370/afm.1768 PMC4369595

[93] Scott LB, Gravely S, Sexton TR, Brzostek S, Brown DL. Effect of patient navigation on enrollment in cardiac rehabilitation. JAMA Internal Medicine. 2013;173(3):244–6. doi: 10.1001/2013.jamainternmed.1042 23247823 10.1001/2013.jamainternmed.1042

[94] Dennis M, O'Rourke S, Slattery J, Staniforth T, Warlow C. Evaluation of a stroke family care worker: results of a randomised controlled trial. BMJ. 1997;314(7087):1071–6; discussion 6–7. Epub 1997/04/12. ; PubMed Central PMCID: PMCPMC2126479. 9133884 10.1136/bmj.314.7087.1071 PMC2126479

[95] Ali-Faisal SF, Benz Scott L, Johnston L, Grace SL. Cardiac rehabilitation referral and enrolment across an academic health sciences centre with eReferral and peer navigation: a randomised controlled pilot trial. BMJ Open. 2016;6(3):e010214 Epub 2016/03/24. doi: 10.1136/bmjopen-2015-010214 ; PubMed Central PMCID: PMCPMC4809077. 27000785 10.1136/bmjopen-2015-010214 PMC4809077

[96] Gardner LI, Metsch LR, Anderson-Mahoney P, Loughlin AM, del Rio C, Strathdee S, et al Efficacy of a brief case management intervention to link recently diagnosed HIV-infected persons to care. AIDS (London, England). 2005;19(4):423–31. Epub 2005/03/08. . 15750396 10.1097/01.aids.0000161772.51900.eb

[97] Wohl AR, Garland WH, Valencia R, Squires K, Witt MD, Kovacs A, et al A randomized trial of directly administered antiretroviral therapy and adherence case management intervention. Clinical infectious diseases: an official publication of the Infectious Diseases Society of America. 2006;42(11):1619–27. Epub 2006/05/03. doi: 10.1086/503906 . 16652320 10.1086/503906

[98] Wohl DA, Scheyett A, Golin CE, White B, Matuszewski J, Bowling M, et al Intensive case management before and after prison release is no more effective than comprehensive pre-release discharge planning in linking HIV-infected prisoners to care: a randomized trial. AIDS and behavior. 2011;15(2):356–64. Epub 2010/11/03. doi: 10.1007/s10461-010-9843-4 ; PubMed Central PMCID: PMCPMC3532052. 21042930 10.1007/s10461-010-9843-4 PMC3532052

[99] Metsch LR, Pereyra M, Messinger S, Jeanty Y, Parish C, Valverde E, et al Effects of a Brief Case Management Intervention Linking People With HIV to Oral Health Care: Project SMILE. American journal of public health. 2015;105(1):77–84. Epub 2014/05/17. doi: 10.2105/AJPH.2014.301871 ; PubMed Central PMCID: PMCPMC4265910. 24832421 10.2105/AJPH.2014.301871 PMC4265910

[100] Metsch L, Feaster D, Gooden L, Matheson T, Stitzer M, Das M, et al Effect of Patient Navigation With or Without Financial Incentives on Viral Suppression Among Hospitalized Patients With HIV Infection and Substance Use: A Randomized Clinical Trial. JAMA. 2016;316(2):156–70. doi: 10.1001/jama.2016.8914 . 27404184 10.1001/jama.2016.8914 PMC5339876

[101] Giordano TP, Cully J, Amico K, R, Davila J, A, Kallen M, A, Hartman C, et al A Randomized Trial to Test a Peer Mentor Intervention to Improve Outcomes in Persons Hospitalized With HIV Infection. Clinical Infectious Diseases. 2016;63(5):678–86. doi: 10.1093/cid/ciw322 . 27217266 10.1093/cid/ciw322 PMC4981755

[102] Bassett IV, Coleman SM, Giddy J, Bogart LM, Chaisson CE, Ross D, et al Sizanani: A Randomized Trial of Health System Navigators to Improve Linkage to HIV and TB Care in South Africa. Journal of Acquired Immune Deficiency Syndromes: JAIDS. 2016;73(2):154–60. doi: 10.1097/QAI.0000000000001025 . 27632145 10.1097/QAI.0000000000001025 PMC5026386

[103] Sullivan C, Leon JB, Sayre SS, Marbury M, Ivers M, Pencak JA, et al Impact of navigators on completion of steps in the kidney transplant process: a randomized, controlled trial. Clinical journal of the American Society of Nephrology: CJASN. 2012;7(10):1639–45. Epub 2012/07/17. doi: 10.2215/CJN.11731111 ; PubMed Central PMCID: PMCPMC3463214. 22798540 10.2215/CJN.11731111 PMC3463214

[104] Navaneethan SD, Jolly SE, Schold JD, Arrigain S, Nakhoul G, Konig V, et al Pragmatic Randomized, Controlled Trial of Patient Navigators and Enhanced Personal Health Records in CKD. Clinical Journal of The American Society of Nephrology: CJASN. 2017;04:04 doi: 10.2215/CJN.02100217 PubMed PMID: 28778854. 10.2215/CJN.02100217 PMC5586570 28778854

[105] Amjad H, Wong SK, Roth DL, Huang J, Willink A, Black BS, et al Health Services Utilization in Older Adults with Dementia Receiving Care Coordination: The MIND at Home Trial. Health Services Research. 2017;12:12 doi: 10.1111/1475-6773.12647 PubMed PMID: 28083879. 10.1111/1475-6773.12647 PMC5785326 28083879

[106] Kneipp SM, Kairalla JA, Lutz BJ, Pereira D, Hall AG, Flocks J, et al Public health nursing case management for women receiving temporary assistance for needy families: a randomized controlled trial using community-based participatory research. American journal of public health. 2011;101(9):1759–68. Epub 2011/07/23. doi: 10.2105/AJPH.2011.300210 ; PubMed Central PMCID: PMCPMC3154225. 21778474 10.2105/AJPH.2011.300210 PMC3154225

[107] Svoren BM, Butler D, Levine BS, Anderson BJ, Laffel LMB. Reducing acute adverse outcomes in youths with type 1 diabetes: A randomized, controlled trial. Pediatrics. 2003;112(4):914–22. . 14523186 10.1542/peds.112.4.914

[108] White VM, Macvean ML, Grogan S, Este CD, Akkerman D, Ieropoli S, et al Can a tailored telephone intervention delivered by volunteers reduce the supportive care needs, anxiety and depression of people with colorectal cancer? A randomised controlled trial. Psychooncology. 2011;21(10):1053–62. Epub 07/20. doi: 10.1002/pon.2019 . 21769989 10.1002/pon.2019

[109] Shaw JM, Young JM, Butow PN, Badgery-Parker T, Durcinoska I, Harrison J, et al Improving psychosocial outcomes for caregivers of people with poor prognosis gastrointestinal cancers: a randomized controlled trial (Family Connect). Supportive Care in Cancer. 2016;24(2):585–95. doi: 10.1007/s00520-015-2817-3 . Language: English. Entry Date: 20160804. Revision Date: 20170601. Publication Type: journal article. Journal Subset: Biomedical. 26111955 10.1007/s00520-015-2817-3

[110] Kneipp SM, Kairalla JA, Lutz BJ, Pereira D, Hall AG, Flocks J, et al Public health nursing case management for women receiving Temporary Assistance for Needy Families: A randomized controlled trial using community-based participatory research. [References]. American Journal of Public Health. 2011;(9):1759–68. doi: 10.2105/AJPH.2011.300210 21778474 10.2105/AJPH.2011.300210 PMC3154225

[111] Thom D, Ghorob A, Hessler D, De Vore D, Chen E, Bodenheimer T. Impact of peer health coaching on glycemic control in low-income patients with diabetes: a randomized controlled trial. Ann Fam Med. 2013;11(2):137–44. doi: 10.1370/afm.1443 . Language: English. Entry Date: 20130920. Revision Date: 20130920. Publication Type: journal article. 23508600 10.1370/afm.1443 PMC3601392

[112] Carrasquillo O, Alonzo Y, Lebron C, Ferras N, Reyes-Arrechea E, Li H, et al A randomized trial of a community health worker led intervention to improve diabetes intermediate outcomes among latinos patients with poorly controlled diabetes. Journal of general internal medicine. 2014;29(23). PubMed PMID: CN-01063665 NEW.

[113] Wohl AR, Garland WH, Valencia R, Squires K, Witt MD, Kovacs A, et al A randomized trial of directly administered antiretroviral therapy and adherence case management intervention. Clinical Infectious Diseases. 2006;42(11):1619–27. doi: 10.1086/503906 . 16652320 10.1086/503906

[114] Lasser KE, Murillo J, Lisboa S, Casimir AN, Shah LV, Emmons KM, et al Colorectal cancer screening among ethnically diverse, low-income patients: A randomized controlled trial. Archives of Internal Medicine. 2011;171(10):906–12. doi: 10.1001/archinternmed.2011.201 . 21606094 10.1001/archinternmed.2011.201

[115] Percac-Lima S, Grant RW, Green AR, Ashburner JM, Gamba G, Oo S, et al A culturally tailored navigator program for colorectal cancer screening in a community health center: a randomized, controlled trial. Journal of General Internal Medicine. 2009;24(2):211–7. doi: 10.1007/s11606-008-0864-x . 19067085 10.1007/s11606-008-0864-x PMC2628981

[116] Weber BE, Reilly BM. Enhancing mammography use in the inner city. A randomized trial of intensive case management. Archives of Internal Medicine. 1997;157(20):2345–9. . 9361575

[117] Wohl DA, Scheyett A, Golin CE, White B, Matuszewski J, Bowling M, et al Intensive Case Management Before and After Prison Release is No More Effective Than Comprehensive Pre-Release Discharge Planning in Linking HIV-Infected Prisoners to Care: A Randomized Trial. AIDS and behavior. 2011;15(2):356–64. doi: 10.1007/s10461-010-9843-4 21042930 10.1007/s10461-010-9843-4 PMC3532052

[118] Paskett E, Tatum C, Rushing J, Michielutte R, Bell R, Foley KL, et al Randomized trial of an intervention to improve mammography utilization among a triracial rural population of women. J Natl Cancer Inst. 2006;98(17):1226–37. Epub 09/07. doi: 10.1093/jnci/djj333 . 16954475 10.1093/jnci/djj333 PMC4450352

[119] Rahm AK, Sukhanova A, Ellis J, Mouchawar J. Increasing utilization of cancer genetic counseling services using a patient navigator model. Journal of Genetic Counseling. 2007;16(2):171–7. doi: 10.1007/s10897-006-9051-6 17277995 10.1007/s10897-006-9051-6

[120] Gardner LI, Metsch LR, Anderson-Mahoney P, Loughlin AM, Rio CD, Strathdee S, et al Efficacy of a brief case management intervention to link recently diagnosed HIV-infected persons to care. Aids. 2005;19(4):423–31. . 15750396 10.1097/01.aids.0000161772.51900.eb

[121] Ali-Faisal SF, Colella TJ, Medina-Jaudes N, Benz Scott L. The effectiveness of patient navigation to improve healthcare utilization outcomes: A meta-analysis of randomized controlled trials. Patient Educ Couns. 2017;100(3):436–48. Epub 2016/10/25. doi: 10.1016/j.pec.2016.10.014 . 27771161 10.1016/j.pec.2016.10.014

