# Supplementary material for: Patient navigators for people with chronic disease: A systematic review
Source: PLoS One. 2018 Feb 20;13(2):e0191980. doi: 10.1371/journal.pone.0191980 (PMC5819768; doi:10.1371/journal.pone.0191980)
Supplement: S1 File — (PDF) [file pone.0191980.s001.pdf]

S1 File. Ovid MEDLINE® search strategy

1. "Continuity of Patient Care"/
2. patient navigation/ or exp patient-centered care/ or case management/
3. ((care or coach* or service* or system*) adj5 (coordinat* or facilitat* or navigat* or transition*)).tw.
4. ((patient* or system* or service*) adj5 (coach* or facilitat* or navigat*)).tw.
5. (guided adj2 care).tw.
6. (case manager* or case management or navigator or navigators).tw.
7. post-discharge support*.tw.
8. 1 or 2 or 3 or 4 or 5 or 6 or 7
9. Chronic Disease/
10. Acquired Immunodeficiency Syndrome/
11. exp Alzheimer Disease/
12. exp Anxiety Disorders/
13. exp Arthritis/
14. exp Asthma/
15. exp Intestinal Diseases/ or exp Irritable Bowel Syndrome/ or exp Colonic Diseases, Functional/ or exp Gastrointestinal Diseases/ or exp Inflammatory Bowel Diseases/ or exp Crohn Disease/ or exp Colitis, Ulcerative/
16. exp Pulmonary Disease, Chronic Obstructive/
17. exp Cerebrovascular Disorders/
18. exp Renal Insufficiency, Chronic/
19. exp Dementia/
20. Depression/
21. exp Depressive Disorder/
22. exp Diabetes Mellitus/
23. exp Emphysema/
24. exp Myocardial Infarction/
25. exp Heart Diseases/
26. exp HIV/ or exp HIV Infections/
27. exp Hypertension/
28. exp Neoplasms/
29. exp Migraine Disorders/
30. exp Mood Disorders/
31. exp Obsessive-Compulsive Disorder/
32. exp Panic Disorder/
33. exp Phobic Disorders/
34. exp Stroke/
35. Ulcer/ or exp peptic ulcer/
36. exp Urinary Incontinence/
37. exp Renal Dialysis/
38. exp Kidney Transplantation/
39. (AIDS or acquired immunodeficiency disorder* or affective disorder* or alzheimer* or agina* or anxiety* or arthritis or asthma or atrial fibrillation or arrhythmia* or bipolar or bladder incontin* or bowel disorder* or brain infarc* or cancer* or

carcinoma* or cardiomyopath* or COPD or COAD or cerebral haemorrhage or cerebral hemorrhage or cerebrovascular disease* or chronic airflow obstruction* or chronic condition* or chronic disease* or chronic illness* or chronic kidney disease* or chronic obstructive airway* disease* or chronic obstructive pulmonary disease* or cognitive impair* or combat disorder* or (coronary adj2 disease*) or crohns or dementia or depressi* or diabetes or diabetic* or dialysis or emphysema or gastrointestinal disease* or haemodialysis or heart arrest or heart attack* or heart disease* or heart failure or heart infarction* or hemodialysis or HIV* or human immunodeficiency virus* or human immuno-deficiency virus* or hypertens* or inflammatory bowel disease* or IBD or irritable bowel* or isch?emia* or insulin-depend* or intracranial h?emorrhage* or intra-cranial h?emorrhage* or kidney transplant* or longterm condition* or long-term condition* or longterm disease* or long-term disease* or longterm illness* or long-term illness* or manic disorder* or migraine* or mood disorder* or myocardial infarc* or neoplasia* or neoplastic or neoplasm* or neurosis or neuroses or neurotic or OCD or obsessive compulsive disorder* or obstructive lung disease* or obstructive pulmonary disease* or PTSD or panic attack* or panic disorder* or phobia* or post-trauma or posttrauma or renal dialysis or stroke or tumor* or tumour* or ulcer or ulcers or ulcerative colitis or urinary incontinence or urination disorder* or ventricular fibrillation).tw.

40. 9 or 10 or 11 or 12 or 13 or 14 or 15 or 16 or 17 or 18 or 19 or 20 or 21 or 22 or 23 or 24 or 25 or 26 or 27 or 28 or 29 or 30 or 31 or 32 or 33 or 34 or 35 or 36 or 37 or 38 or 39
41. 8 and 40
42. (randomized controlled trial or controlled clinical trial).pt.
43. drug therapy.sh.
44. (groups or placebo or randomized or randomly or trial).tw.
45. 42 or 43 or 44
46. 41 and 45
47. (animals not humans).sh.
48. 46 not 47
