# Supplementary material for: Patient navigators for people with chronic disease: A systematic review
Source: PLoS One. 2018 Feb 20;13(2):e0191980. doi: 10.1371/journal.pone.0191980 (PMC5819768; doi:10.1371/journal.pone.0191980)
Supplement: S1 Table — (PDF) [file pone.0191980.s003.pdf]

S1 Table. Risk of bias by individual study

Study<br><br>First Author (Year)	Random sequence generation	Allocation concealment	Blinding of outcome assessment	Incomplete outcome data	Group similarity at baseline	Intention-to-treat analysis
**Cancer screening**						
Weber (1997)						
West (2004)						
Paskett (2006)						
Rahm (2007)						
Ahmed (2010)						
Phillips (2011)						
Marshall (2015)						
Taylor (2002)						
Taylor (2010)						
Basch (2006)						
Jandorf (2005)						
Christie (2008)						
Percac-Lima (2008)						
Coronado (2011)						
Lasser (2011)						
Green (2013)						
Myers (2012)						
Myers (2014)						
Ritvo (2015)						
Dietrich (2006)						
Braun (2015)						
Enard (2015)						
Percac-Lima (2016)						
Greenspan (2016)						
Cole (2017						
DeGroff (2017)						
Guillame (2017)						

Study<br><br>First Author (Year)	Random sequence generation	Allocation concealment	Blinding of outcome assessment	Incomplete outcome data	Group similarity at baseline	Intention-to-treat analysis
**Cancer diagnosis**						
Ell (2007)						
Ferrante (2007)						
Crump (2008)						
Bastani (2010)						
Giese-Davis (2016)						
Maxwell (2010)						
Engelstad (2005)						
Miller (1997)						
Lerman (1992)						
Paskett (2012)						
Raich (2012)						
Wells (2012)						
**Cancer treatment**						
White (2011)						
Ell (2009)						
Fiscella (2012)						
Percac-Lima (2015)						
Shaw (2016)						
**Diabetes**						
Corkery (1997)						
Laffel (1998)						
Gary (2003)						
Svoren (2003)						
Spencer (2011)						
Thom (2013)						
Prezio (2013)						
Carrasquillo (2014)						
**HIV**						

Study<br>First Author (Year)	Random sequence generation	Allocation concealment	Blinding of outcome assessment	Incomplete outcome data	Group similarity at baseline	Intention-to-treat analysis
Gardner (2004)	Unclear	Low	Low	Low	Unclear	Low
Wohl (2006)	Low	Low	Low	Low	Low	Low
Wohl (2011)	Unclear	Unclear	Low	Low	Low	Low
Metsch (2015)	Low	Low	Unclear	Low	Low	Unclear
Metsch (2016)	Low	Low	Unclear	Low	Low	Low
Giordano (2016)	Low	Low	Low	Low	Low	Low
Bassett (2016)	Low	Low	Low	Low	Low	Low
**Cardiovascular**						
Dennis (1997)	Low	Low	Low	Low	Low	Low
Scott (2013)	Low	Unclear	Low	High	Low	Unclear
Willard-Grace (2015)	Unclear	Low	Unclear	Low	Low	Low
Ali-Faisal (2016)	Low	Low	Low	Unclear	Low	Unclear
**Chronic kidney disease**						
Sullivan (2012)	Low	Unclear	Unclear	Low	Low	Low
Navaneethan (2017)	Low	Low	Unclear	Unclear	Low	Unclear
**Dementia**						
Amjad (2017)	Unclear	Unclear	Low	High	Unclear	Low
**Multiple**						
Kneipp (2011)	Low	Low	Low	Low	Low	Unclear

Low risk of bias

Unclear risk of bias

High risk of bias
