# Supplementary material for: Patient navigators for people with chronic disease: A systematic review
Source: PLoS One. 2018 Feb 20;13(2):e0191980. doi: 10.1371/journal.pone.0191980 (PMC5819768; doi:10.1371/journal.pone.0191980)
Supplement: S2 Table — (PDF) [file pone.0191980.s004.pdf]

S2. Attributes of patient navigator interventions

Author	Year	Navigator type				Intervention features												Communication medium					Frequency			Duration			
		Lay	Peer	Professional	Not reported	Facilitates care	Schedules appointments	Accompaniment	Practical assistance	Language	Culturally tailored	Health literacy	Attitudes and beliefs	Promotes adherence	Reminders	Education	Psychosocial	Phone	In-person - patient home	In-person - other	Mail	E-mail	Brief	Medium	High	<=3 months	3-12 months	>12 months	Significant outcome
**Cancer treatment**																													
Fiscella/Hendren	2012	■				■	■	■	■	■		■	■	■	■	■	■	■		■	■	■			■		■		
Ell	2009	■				■			■	■	■	■	■	■		■	■	■		■					■		■		
White	2011	■				■							■				■	■			■			■			■		■
Percac-Lima	2015	■				■				■		■			■			■					■			■			■
Giese-Davis	2016		■			■					■						■		■		■				■		■		■
Shaw	2016			■		■			■							■	■	■						■		■			
**Cancer diagnosis**																													
**Breast cancer**																													
Ferrante	2007	■				■	■		■		■	■			■		■	■		■				■		■			■
Ell	2007	■		■		■				■			■		■	■	■	■						■			■		■
Crump	2008	■				■		■	■		■		■		■	■	■	■		■	■				■		■		■
Maxwell	2010		■				■	■	■	■	■	■			■	■	■	■	■	■					■		■		■
Bastani	2010	■		■			■		■		■		■		■	■	■	■			■				■		■		
**Cervical cancer**																													
Lerman	1992			■			■		■				■			■	■	■					■			■			■

Author	Year	Navigator type				Intervention features												Communication medium					Frequency			Duration			
		Lay	Peer	Professional	Not reported	Facilitates care	Schedules appointments	Accompaniment	Practical assistance	Language	Culturally tailored	Health literacy	Attitudes and beliefs	Promotes adherence	Reminders	Education	Psychosocial	Phone	In-person - patient home	In-person - other	Mail	E-mail	Brief	Medium	High	<=3 months	3-12 months	>12 months	Significant outcome
Miller	1997			■					■				■			■	■	■					■			■			■
Engelstad	2005	■					■				■	■	■		■	■	■	■	■	■				■			■		■
**Multiple types**																													
Wells/Lee	2012	■				■			■	■	■	■	■			■	■	■	■	■					■		■		
Raich	2012	■				■	■	■		■		■	■			■	■	■	■	■		■			■		■		■
Paskett	2012	■				■	■		■	■			■			■	■	■		■					■			■	■
**Cancer screening**																													
**Breast cancer**																													
Weber	1997	■					■	■	■	■	■		■		■	■	■	■	■	■	■				■		■		■
West	2004	■							■		■		■		■	■	■	■			■		■			■			
Paskett	2006	■					■		■		■	■	■		■	■	■	■	■		■			■			■		■
Rahm	2007	■					■								■	■		■			■			■		■			
Ahmed	2010	■									■		■		■	■	■			■	■		■			■			■
Phillips	2011	■				■	■		■	■	■		■			■	■	■			■			■			■		■
Marshall	2015	■					■	■			■	■	■			■		■		■	■				■			■	■
**Cervical cancer**																													
Taylor	2002	■					■		■	■	■	■	■			■	■	■	■		■		■			■			■
Taylor	2010	■								■	■	■	■			■	■	■	■		■		■			■			

Author	Year	Navigator type				Intervention features												Communication medium					Frequency			Duration			
		Lay	Peer	Professional	Not reported	Facilitates care	Schedules appointments	Accompaniment	Practical assistance	Language	Culturally tailored	Health literacy	Attitudes and beliefs	Promotes adherence	Reminders	Education	Psychosocial	Phone	In-person - patient home	In-person - other	Mail	E-mail	Brief	Medium	High	<=3 months	3-12 months	>12 months	Significant outcome
**Colorectal cancer**																													
Jandorf	2005	■					■				■				■	■		■			■				■		■		■
Basch	2006				■				■				■			■	■	■							■		■		■
Percac-Lima	2008	■					■	■	■	■	■	■	■		■	■	■	■		■	■				■		■		■
Christie	2008				■	■	■		■	■			■		■	■		■			■			■			■		
Lasser	2011	■				■	■	■	■	■		■	■			■	■	■			■				■		■		■
Coronado	2011	■								■	■	■	■		■	■		■	■		■		■				■		
Green	2013			■		■	■					■	■		■	■		■			■	■	■					■	■
Myers/Lairson	2012			■		■							■		■	■		■			■		■			■			■
Myers	2014				■	■						■	■		■	■		■			■		■			■			■
Enard	2015	■				■	■			■		■			■	■	■	■			■				■			■	■
Ritvo	2015			■		■	■					■	■			■		■		■	■		■			■			■
Greenspan	2016	■											■			■		■					■			■			
Cole	2017		■			■			■		■		■			■		■					■				■		■
Guillame	2017			■								■			■	■		■	■		■		■			■			■
DeGroff	2017	■				■	■	■	■	■			■		■	■	■	■		■	■		■				■		■
**Multiple types**																													

Author	Year	Navigator type				Intervention features												Communication medium					Frequency			Duration			
		Lay	Peer	Professional	Not reported	Facilitates care	Schedules appointments	Accompaniment	Practical assistance	Language	Culturally tailored	Health literacy	Attitudes and beliefs	Promotes adherence	Reminders	Education	Psychosocial	Phone	In-person - patient home	In-person - other	Mail	E-mail	Brief	Medium	High	<=3 months	3-12 months	>12 months	Significant outcome
Dietrich	2006	■					■		■	■		■	■		■	■	■	■			■				■			■	
Braun	2015	■				■	■		■		■	■			■	■		■	■		■			■				■	■
Percac-Lima	2016	■					■	■	■	■			■	■	■	■	■	■									■		■
**Diabetes**																													
Corkery	1997	■				■	■	■	■	■	■				■					■					■		■		■
Laffel	1998	■					■		■	■					■			■		■	■				■			■	■
Svoren	2003	■					■		■	■			■		■	■		■		■	■				■			■	■
Gary	2003	■				■	■				■			■		■	■	■	■		■				■			■	
Spencer	2011	■				■		■		■	■	■				■		■	■	■				■			■		■
Thom	2013		■			■				■	■			■		■	■	■		■					■		■		■
Prezio	2013	■				■					■	■		■		■				■					■		■		■
Carrasquillo	2014	■							■							■	■	■	■	■					■		■		■
**Cardiovascular risk**																													
Willard-Grace/Thom	2015	■				■	■	■		■	■	■	■	■		■	■	■		■					■		■		■
**Cardiovascular disease**																													
Dennis	1997			■		■			■								■	■		■					■		■		
Scott/Scott	2013	■				■									■	■		■		■	■		■			■			■

Author	Year	Navigator type				Intervention features												Communication medium					Frequency			Duration			
		Lay	Peer	Professional	Not reported	Facilitates care	Schedules appointments	Accompaniment	Practical assistance	Language	Culturally tailored	Health literacy	Attitudes and beliefs	Promotes adherence	Reminders	Education	Psychosocial	Phone	In-person - patient home	In-person - other	Mail	E-mail	Brief	Medium	High	<=3 months	3-12 months	>12 months	Significant outcome
Ali-Faisal	2016		■										■	■		■		■		■	■		■			■			
**HIV/AIDS**																													
Gardner	2004				■	■		■	■							■	■	■		■				■		■			■
Wohl	2006				■	■			■					■						■					■		■		
Wohl	2011				■	■			■									■		■					■		■		
Metsch	2015				■	■	■		■				■			■			■	■				■		■			■
Metsch	2016	■				■	■	■	■				■	■	■	■	■		■	■					■			■	
Giordano	2016		■							■		■	■	■		■	■	■		■				■			■		
Bassett	2016	■										■		■	■	■	■	■		■		■		■			■		
**Chronic kidney disease**																													
Sullivan	2012		■			■			■			■	■		■	■	■			■					■			■	■
Navaneethan	2017	■				■	■	■	■					■		■	■	■		■		■			■			■	
**Dementia**																													
Amjad	2017	■				■		■	■				■	■	■	■	■	■	■						■			■	
**Multiple chronic diseases**																													
Kneipp	2011		■		■			■		■					■	■	■	■	■					■		■		■	
